# Targeting Pulmonary Hypertension: Elucidating Sophocarpine’s Protective Role via Preclinical Models

**DOI:** 10.1155/mi/5524066

**Published:** 2026-02-23

**Authors:** Feng Xie, Jie Feng, Kai Li, Yi Chen, Leilei Han, Yanqing Wu

**Affiliations:** ^1^ Department of Cardiology, The Second Affiliated Hospital, Jiangxi Medical College, Nanchang University, Nanchang, 330006, China, ncu.edu.cn; ^2^ Jiangxi Key Laboratory of Molecular Medicine, The Second Affiliated Hospital of Nanchang University, Nanchang, 330006, China, jxndefy.cn

**Keywords:** apoptosis, inflammation, pulmonary hypertension, sophocarpine, vascular remodeling

## Abstract

**Background:**

Pulmonary hypertension (PH) is a serious disease that manifests itself as elevated pressure within the pulmonary arteries. The onset of PH is usually insidious, and if left untreated, it may lead to heart failure and even life‐threatening conditions. Recent studies have shown that sophocarpine (SOP) has significant effects on antioxidant, anti‐inflammatory, antifibrotic, and hemodynamic improvement, and it may become an emerging drug for the treatment of PH. However, the specific mechanism of action of SOP still requires further experimental validation.

**Methods:**

We established in vivo and in vitro models of PH and treated them with varying concentrations of SOP. To visualize the changes in rats and cells, we used scratch assay, flow cytometry, Western blotting, pulmonary artery pressure measurement, biochemical analysis, enzyme‐linked immunosorbent assay (ELISA), ultrasound scanning, hematoxylin and eosin (HE) staining, and Masson’s trichrome staining.

**Results:**

Our results showed that SOP significantly alleviated the inflammatory response and apoptosis induced by PH, reduced pulmonary artery pressure, and restored the balance of pulmonary artery remodeling. These effects were found to be effective in alleviating PH.

**Conclusion:**

Our study provides clear evidence that SOP has a significant protective effect in the PH model and is expected to be a promising therapeutic agent for PH.

## 1. Introduction

Pulmonary hypertension (PH) is a pathological condition characterized by elevated pulmonary artery pressure. Typically, this is quantified by right heart catheterization, with a mean pulmonary artery pressure (mPAP) exceeding 20 mmHg at rest [1, 2]. PH represents a significant global health challenge, affecting individuals across all age demographics, with current estimates indicating a prevalence of ~1% within the global population [3]. The incidence of PH is particularly pronounced among those over 65, due to the involvement of both cardiac and pulmonary factors [4]. The global incidence of PH is estimated to be between 15 and 50 cases per 100,000 people, increasing with age. A recent study in the United Kingdom has highlighted a disturbing trend, with the prevalence of PH having doubled over the past decade, now standing at 125 cases per million residents [5]. The prognosis for individuals with PH is grim, particularly in the absence of treatment. Advanced right heart failure remains the principal cause of mortality in patients with primary PH. Recent data suggests that the 5‐year survival rate for patients with pulmonary arterial hypertension (PAH) in Europe hovers around 61%, with survival rates markedly diminishing as the disease progresses and clinical manifestations intensify [6]. The insidious onset of PH, with early symptoms such as dyspnea and malaise that frequently overlap with those of other conditions, often leads to a diagnosis at an advanced stage. Consequently, the risk of mortality increases substantially within the first 2 years of diagnosis, with many patients succumbing within 1–3 years of being diagnosed [7]. Currently, therapeutic strategies for PH include endothelin receptor antagonists, phosphodiesterase‐5 inhibitors, and prostacyclin analogs, all of which have led to notable improvements in survival rates—from a mere 34% 5‐year survival rate in 1991 to over 60% in 2015 [8]. The recent approval of sotatercept marks a significant advancement in the pharmacological management of PH [9]. However, while existing treatments offer symptomatic relief, alleviate vasoconstriction, and mitigate heart failure, they do not provide a cure and fail to eliminate the disease’s inherent lethality. Thus, the continued development of novel therapeutic agents remains a critical avenue for advancing the management of PH.

Sophocarpine (SOP), a natural alkaloid and bioactive component obtainable from various Chinese medicinal plant species, exhibits potent anti‐inflammatory, immunomodulatory, and antioxidant properties [10]. In recent years, SOP has garnered considerable attention from researchers due to its promising potential in immunomodulation. Its distinctive capacity to attenuate inflammatory responses and enhance immune function positions it as a compelling biotherapeutic candidate for the management of PH. SOP has been shown to modulate immune system equilibrium by inhibiting the secretion of pro‐inflammatory cytokines and amplifying anti‐inflammatory responses [11, 12]. It impedes the proliferation and migration of endothelial cells within the carotid vasculature by curbing inflammatory responses, which may, in turn, slow the pathological remodeling of pulmonary arteries and reduce pulmonary artery pressure. These effects hold significant therapeutic promise for enhancing cardiac function in PH patients. Moreover, SOP can exert an inhibitory influence on the fibrotic process [13]. By reducing the deposition of extracellular matrix components, SOP can alleviate lung tissue fibrosis and prevent further pulmonary damage, thus helping to preserve lung function in patients with PH. However, there is still a lack of in‐depth research into its exact role in regulating inflammation, oxidative stress, apoptosis and vascular remodeling in PH [14].

## 2. Materials and Methods

### 2.1. Materials

40 male wild‐type (WT) Sprague‐Dawley (SD) rats, aged 6–8 weeks and weighing 200 ± 20 g, were procured from Nanjing Corues Animal Company. SOP (CAS Number HY‐N0103), monocrotaline (MCT, Cat Number HY‐N0750), and platelet‐derived growth factor (PDGF‐BB, CAS Number HY‐P73351) were bought from MedChemExpress (MCE, New Jersey, USA). The flow cytometry apoptosis kit was purchased from Beyotime (Number C1062S, Shanghai, China). Primary antibodies targeting GAPDH, β‐tubulin, Bax, Bcl‐2, α‐SMA, vimentin, IL‐1β, iNOS, Toll‐like receptor 4 (TLR4), and Superoxide Dismutase 1 (SOD‐1) were acquired from Proteintech (Proteintech Group, Inc., USA), while antibodies specific to NRF2 and HO‐1 were obtained from ABclonal Biotechnology Co., Ltd. (ABclonal, China). Anti‐cleaved caspase‐3 antibody (ab32042) was purchased from abcam (UK).

### 2.2. Rats Treatments

The rats utilized in this investigation were housed in the Animal Center at Nanchang University, where they underwent a 1‐week acclimatization period to the housing conditions. The ambient temperature and humidity were carefully regulated at 23 ± 2°C and 53 ± 2%, respectively. We selected the concentration of SOP to be administered based on the results of previous study [15, 16]. A total of 40 rats were randomly assigned to four groups using a computer‐generated random number table (GraphPad Prism 9.0) by an independent statistician not involved in subsequent experimental procedures (Figure 1A): Control group (*n* = 10): Rats in this group received a single intraperitoneal injection of saline, followed by daily saline (2 mL) injections starting 3 weeks after the initial injection and continuing for 2 weeks. MCT group (*n* = 10): Rats in the MCT group were intraperitoneally injected with MCT at a dose of 60 mg/kg. After a 3‐week interval, they received daily intraperitoneal saline (2 ml) injections for two consecutive weeks. MCT + SOP (20 mg/kg) (*n* = 10): These rats were initially injected with MCT (60 mg/kg), followed by a second injection of SOP at 20 mg/kg (dissolved in 2 mL of saline solution), administered once daily for 2 weeks, starting 3 weeks after the MCT injection. MCT + SOP (40 mg/kg) (*n* = 10): Following the initial MCT injection (60 mg/kg), rats in this group were treated with SOP (40 mg/kg, dissolved in 2 mL of saline solution) intraperitoneally once daily for 2 weeks, beginning 3 weeks after the MCT injection. Group concealment is achieved by sealing group information corresponding to animal ID numbers within opaque sequentially numbered envelopes. Only technicians responsible for initial dosing may access these envelopes, which are opened immediately prior to administration. After grouping, all animals are fitted with unique ear tags to replace group identifiers, ensuring operators remain unaware of animal group assignments after initial dosing.

Figure 1Schematic diagram of the pulmonary hypertension animal model experimental workflow, screening of sophocarpine concentrations, and cell scratch assay. (A) Schematic diagram of the pulmonary arterial hypertension animal model establishment and experimental workflow. (B) CCK8 assay to detect cell activity to screen the appropriate Sophocarpine drug concentration (*n* = 6 per group). (C–D) Microscopic diagram and statistical histogram of cell scratch assay (*n* = 3 per group). (E) Detect the effects of administering the SOP at 24 and 48 h on the HPASMC proliferation assay (*n* = 3 per group). Data are expressed as mean ± sd.

**Figure:**
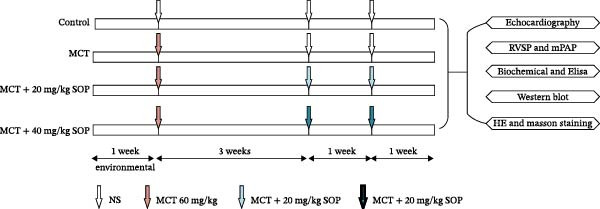
(A)

**Figure:**
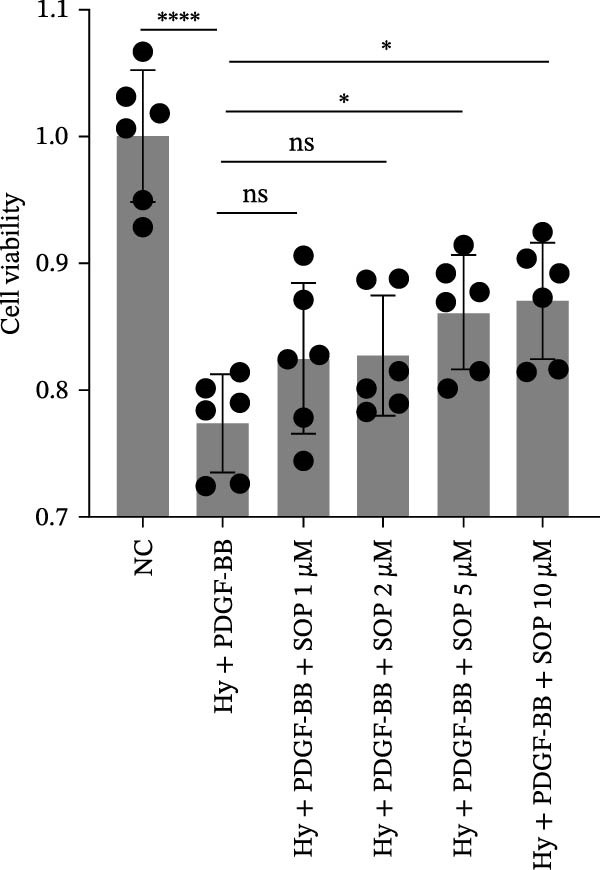
(B)

**Figure:**
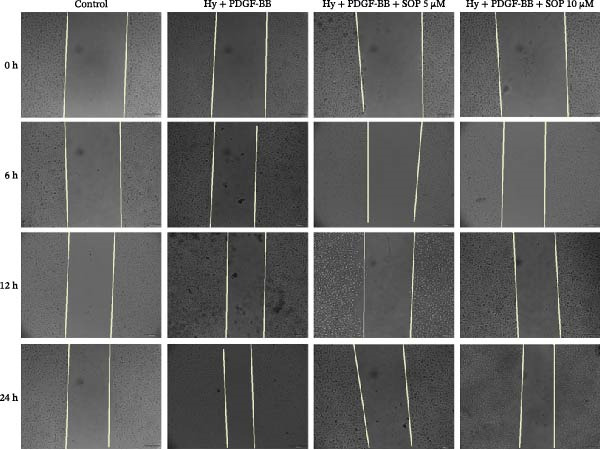
(C)

**Figure:**
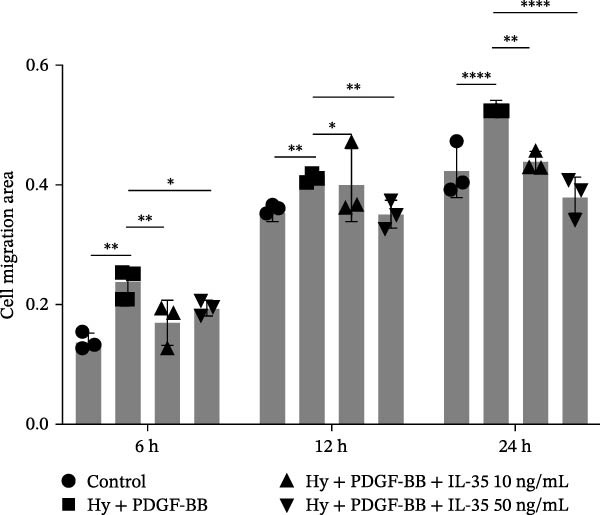
(D)

**Figure:**
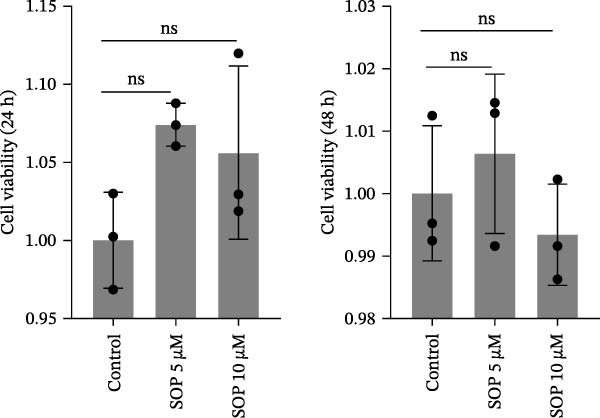
(E)

### 2.3. Cell Culture and Treatment and Cell Counting Kit‐8 (CCK8) Assay

Human pulmonary artery smooth muscle cells (HPASMCs) were purchased from Otwo Biotech (Catalog Number HTX2164, Guangzhou, China). These cells were cultured in a specialized smooth muscle cell medium at 37°C in a 5% CO_2_ incubator. A PH model was constructed by treating the cells with 1% O_2_ hypoxia combined with PDGF‐BB (20 ng/mL) for 24 h [17]. The appropriate SOP drug concentration was selected based on CCK8 assay results.

We evaluated the effect of SOP on cell viability using the CCK8 assay to determine the appropriate concentration for cell treatment. HPASMCs were divided into six groups: (1) normoxia control (no treatment); (2) hypoxia (Hy) + PDGF‐BB (20 ng/mL); (3) Hy + PDGF‐BB + SOP 1 μM; (4) Hy + PDGF‐BB + SOP 2 μM; (5) Hy + PDGF‐BB + SOP 5 μM; (6) Hy + PDGF‐BB + SOP 10 μM.

The cells were seeded into 96‐well plates at a density of 1 × 10^4^ cells per well (100 μL per well) and incubated overnight to allow attachment. The cells were then treated with PDGF‐BB and SOP under hypoxic conditions (1% O_2_, 5% CO_2_, 37°C) for 24 h. After treatment, the medium was removed, and 100 μL of fresh medium containing 10 μL of CCK8 reagent was added to each well. The plates were then incubated for an additional 2–3 h and the absorbance at 450 nm was measured using a microplate reader. Cell viability was calculated relative to the normoxia control group, and two optimal SOP concentrations were selected for subsequent experiments. Based on the results of the CCK8 assay, 5 μM and 10 μM were selected as the recommended SOP concentrations for the subsequent cell experiments.

### 2.4. Echocardiography

An echocardiographic assessment was performed by a veterinary sonographer unaware of the grouping information. General anesthesia was induced using isoflurane, with the depth of anesthesia carefully adjusted to ensure stable respiration and heart rate. The anesthetized animals were positioned supine on the ultrasound table, ensuring that their posture remained secure throughout the procedure. An animal respirator was then connected, and the fur over the rats’ thoracic region was shaved to facilitate clearer imaging. A suitable amount of ultrasound coupling gel was applied to the area of contact between the probe and the skin, ensuring optimal transmission of sound waves. The ultrasound probe was positioned slightly to the left of the midline of the rats’ chest to enable visualization of both the left and right ventricles. The probe was occasionally tilted abdominally to capture a subcostal view of the heart. The acquired images were subsequently processed and analyzed using specialized software to quantify various cardiac parameters, including right ventricular (RV) fractional area change (RVFAC), tricuspid annular plane systolic excursion (TAPSE), and end‐diastolic right ventricular free wall thickness (RVFWT). Upon completion of the imaging procedure, the rats were closely monitored during the recovery phase to ensure full reversal of anesthesia, while their heart rate, respiratory function, and motor activity were diligently assessed. Ultrasound images and video clips were stored as encrypted files within a password‐protected folder and analyzed offline by two trained researchers unaware of the grouping. They performed TAPSE, RVFAC, and RVFWT measurements on rats without knowledge of each other’s results. Variability was quantified using CV and ICC (two‐way mixed‐effects model, absolute agreement)—ICC values >0.9 indicate excellent agreement, while 0.75–0.9 indicate good agreement.

### 2.5. Measurement of RV Systolic Pressure (RVSP)and mPAP

Right heart catheterization was performed to measure RVSP and mPAP. First, general anesthesia was induced using 2% isopentobarbital to ensure the rats remained pain‐free and motionless throughout the procedure. The animals were then positioned supine on the dissection table, and the right external jugular vein was meticulously isolated from the right side of the neck. Approximately 1 cm of the vein was freed, with the distal end ligated and a loosely tied knot applied to the proximal end for secure backup. The proximal and distal ends of the vein were anchored to the chest and neck skin using hemostatic forceps to ensure the vein was fully exposed and appropriately distended. The biofunctional experimental system was then calibrated to maintain the sensor in parallel alignment with the position of the rat’s heart. A V‐shaped incision was made at the proximal end of the heart using ophthalmic scissors, and the curved tip of the right heart catheter was inserted into the vascular incision. A live knot was employed to secure the catheter in place temporarily. The catheter was then gently advanced, initially 1–2 cm to reach the superior vena cava, followed by an additional 2–3 cm to enter the right atrium. Progressing with slight rotational movements, the catheter was advanced further, ~3.5–4 cm, until it reached the right ventricle. At this point, the catheter was paused briefly before being gently advanced into the pulmonary artery. During this procedure, the position of the catheter tip was carefully monitored, with the shifting pressure curve displayed on the monitor used as a guide for proper placement. RVSP and mPAP were then recorded using a right jugular vein right heart catheter (ADV500 Pressure‐Volume System, Transonic). Blinded procedures were maintained throughout the entire data collection and analysis process to minimize observer bias. Neither the technicians performing catheter insertions nor the researchers analyzing pressure curves were aware of the group assignments.

### 2.6. Tissue Sampling in Rats

All rats were initially weighed and subsequently anesthetized with sodium pentobarbital (30 mg/kg). Following spinal cord dislocation, blood, heart, and lung tissues were swiftly harvested. Tissue samples from both the heart and lungs were fixed in 4% paraformaldehyde and immersed in water, secured in place using cotton balls to ensure complete submersion. The samples were then dehydrated and processed for paraffin embedding. After these preparatory steps, any residual liquid adhering to the surface of the tissue samples was carefully removed. The remaining lung lobes were dissected along their anatomical segments, cut into small tubular sections, and preserved at −80°C for subsequent tissue protein extraction. To facilitate morphological examination and accurate weighing, the large blood vessels surrounding the heart were carefully trimmed. The atria and ventricles were separated at the atrioventricular junction, and the free wall of the right ventricle was clamped at the junction between the right ventricle and septum. Any adhesive fluid within the tissue was expelled for further morphological comparison. The Fulton’s Index was calculated using the formula: RV weight / (left ventricular + septal weight), denoted as RV / (LV + S).

### 2.7. Biochemical and Enzyme‐Linked Immunosorbent Assay (ELISA)

The rat blood samples were subjected to centrifugation, and the resulting serum was carefully collected. The levels of malondialdehyde (MDA) and the activity of SOD were subsequently quantified using commercially available biochemical assays (Shanghai Enzyme‐linked Biotechnology Co., Ltd.). Additionally, the concentrations of IL‐1β, IL‐6, and TNF‐α in the serum were measured using ELISA kits (Jiangsu Meimian Industry Co., Ltd.).

### 2.8. Hematoxylin‐Eosin (HE) and Masson’s Trichrome Staining

The lung and RV tissues were thoroughly rinsed three times with ice‐cold saline. A segment of the aortic tissue was then fixed in a 4% formaldehyde solution for 1 h, following which it was sectioned into 5 μm slices at varying depths. These sections were subsequently subjected to HE staining and Masson’s trichrome staining according to established protocols. Stained sections were scanned and submitted to board‐certified pathologists for qualitative assessment, followed by quantitative morphometric analysis using ImageJ software by researchers unaware of the grouping.

### 2.9. Wound Healing Assay

To test the wound healing capacity of a PH cell model, we performed scratch experiments on HPASMCs. Once the cell density had reached 80%, the HPASMCs were scratched using a 200 μL pipette tip. The cells were then incubated in serum‐free medium and photographed after 0, 6, 12, and 24 h, respectively. ImageJ software was used to analyze the data.

### 2.10. Detection of Apoptosis by Flow Cytometry

To detect apoptosis in the PH cell model, we performed a flow cytometry analysis of HPASMCs. Once the HPASMC cell density reached 70%–80%, the cells were divided into four groups as described above. After 24 h of treatment, the cells were harvested. After washing, the cells were resuspended in 195 μL of binding buffer at a concentration of 1 × 10^6^ cells/mL. Five microlitres of Annexin V‐FITC and five microlitres of propidium iodide (PI) were then added to the cell suspension. The mixture was gently mixed and incubated in the dark for 15 min at room temperature. After incubation, 300 μL of binding buffer was added to each sample. The stained cells were analyzed using a flow cytometer (BD FACSCalibur, BD, USA), exciting both dyes at 488 nm and detecting the emission at 530 nm (Annexin V‐FITC) and 620 nm (PI), respectively. Ten thousand events per sample were collected and analyzed using CytExpert software (Beckman Coulter, version 2.6) to quantify the ratios of early apoptotic cells, late apoptotic cells, and necrotic cells.

### 2.11. Western Blot Analysis

Total protein was extracted from rat lung tissue and HPASMCs, and protein concentrations were quantified using a BCA protein assay kit (Cat Number PA115, Tiangen, Beijing, China). A total of 20 μg of protein per sample was subjected to separation by 10% sodium dodecyl sulfate‐polyacrylamide gel electrophoresis (SDS‐PAGE), followed by transfer onto polyvinylidene difluoride (PVDF) membranes (Millipore, Bedford, MA, USA). The membranes were subsequently blocked with 5% skimmed milk for 60 min. After blocking, the membranes were incubated overnight with primary antibodies at the following dilutions: anti‐Bax (1:1000), anti‐Bcl‐2 (1:1000), anti‐α‐SMA (1:1000), anti‐vimentin (1:100), anti‐iNOS (1:1000), anti‐SOD‐1 (1:1000), anti‐IL‐1β (1:1000), anti‐TLR4 (1:1000), anti‐NRF2 (1:1000), anti‐HO‐1 (1:1000), anti‐GAPDH (1:5000), anti‐cleaved caspase‐3 (1:500), and anti‐β‐tubulin (1:5000). The membranes were then incubated with appropriate secondary antibodies [18]. Immunoblotting was performed using the relevant antibodies, and protein bands were visualized and analyzed using the Image Lab V 6.1 software (Bio‐Rad Laboratories, Life Science, CA, USA). The technicians performing the tests and the researchers analyzing the results were unaware of the group assignments.

## 3. Statistical Analysis

All experiments were performed in triplicate biological replicates. Statistical analysis was conducted using GraphPad Prism 9.0.2 software (GraphPad Software Inc., San Diego, CA, USA). Data normality was assessed using the Shapiro–Wilk test, and homogeneity of variance was confirmed via Levene’s test. Differences between groups were analyzed using the unpaired Student’s *t*‐test. Multiple comparisons were performed using one‐way analysis of variance (ANOVA), followed by Tukey’s post hoc test. Data are presented as the mean ± standard deviation (SD), with *p*‐values of <0.05 considered statistically significant.

## 4. Results

### 4.1. SOP Enhanced Cell Activity but Inhibited Cell Migration

A CCK8 assay of cell viability showed that viability was significantly lower in the Hy+ PDGF‐BB group than in the control group (*p* < 0.01). However, SOP treatment increased viability in a statistically significant manner at concentrations of 5 μM and 10 μM (Figure 1B). Additionally, wound repair experiments revealed that the migratory capacity of Hy+ PDGF‐BB‐treated cells was notably higher than that of cells in the control group, in line with prior observations. Conversely, SOP was found to reduce the migratory capacity of cells (Figure 1C,D). To eliminate the interference of proliferation on migration outcomes, we employed the CCK8 assay to investigate the effect of SOP on cell proliferation under normoxic conditions. As shown in Figure 1E, under serum‐free conditions, SOP did not promote HPASMC proliferation at either 24 or 48 h of culture, validating the reliability of the scratch assay results.

### 4.2. SOP Inhibits MCT‐Induced Oxidative Stress and Inflammatory Responses in PH Rats

Inflammatory responses and oxidative stress in rats were evaluated through biochemical assays and ELISA. As depicted in Figure 2, compared to the control group, the MCT group exhibited markedly elevated levels of inflammatory mediators, including IL‐1β, IL‐6, and TNF‐α, along with a significant increase in the expression of SOD activity and MDA. Notably, the expression of these biomarkers was considerably reduced in the MCT + SOP groups when compared to the MCT group.

Figure 2Changes in inflammation and oxidative stress in groups of experimental rats using ELISA and biochemical assays. (A–C) The concentrations of IL‐1β, IL‐6, and TNF‐α in the serum were measured using ELISA kits. (D, E) The levels of MDA and the activity of SOD using commercially available biochemical assays. Data are expressed as mean ± sd, *n* = 3 per group.

**Figure:**
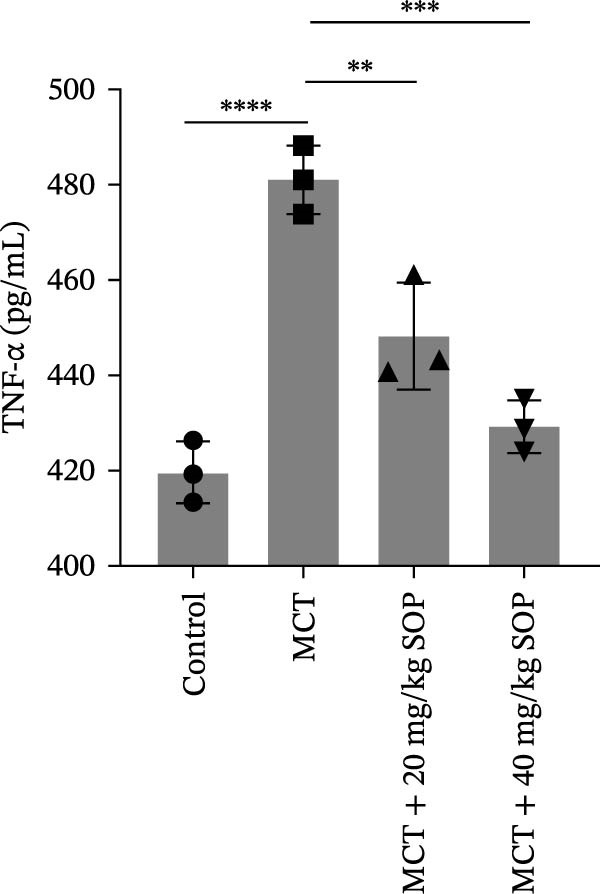
(A)

**Figure:**
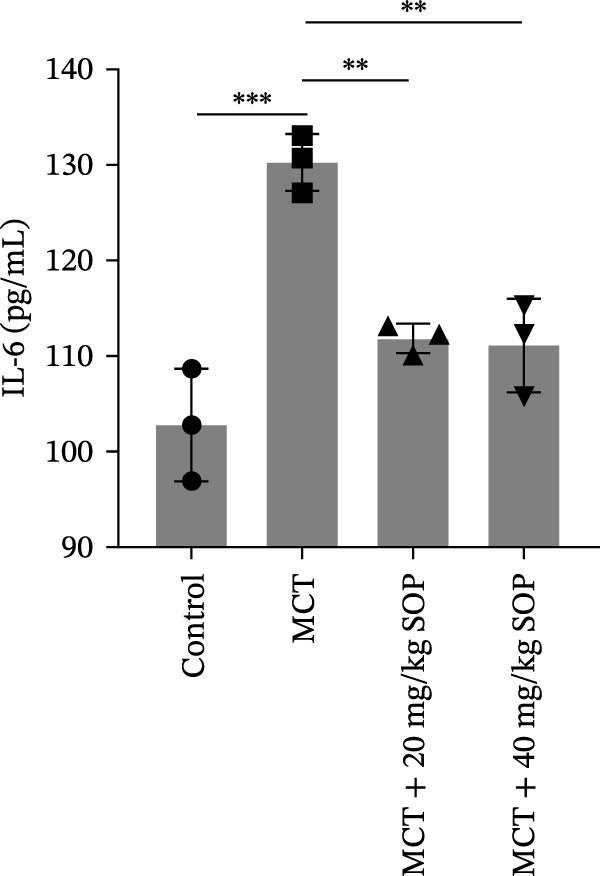
(B)

**Figure:**
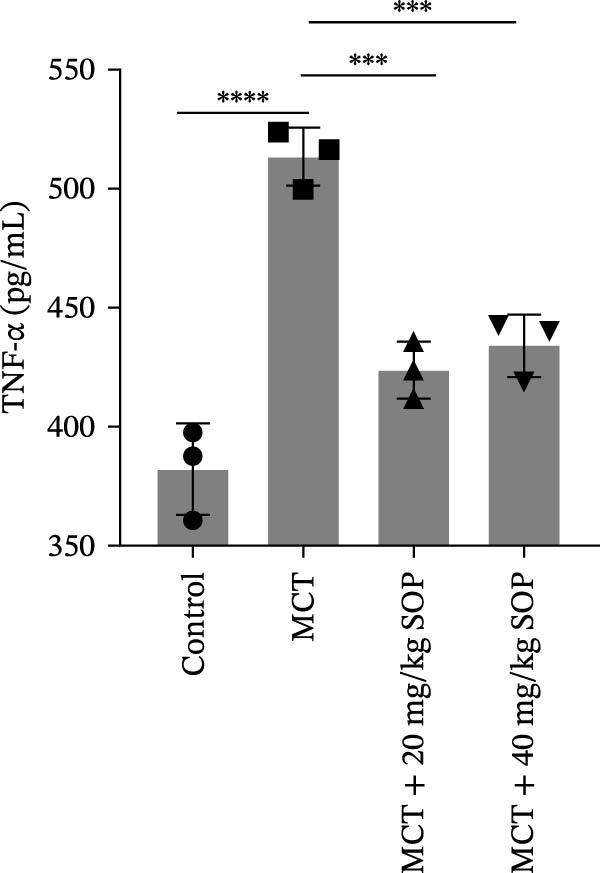
(C)

**Figure:**
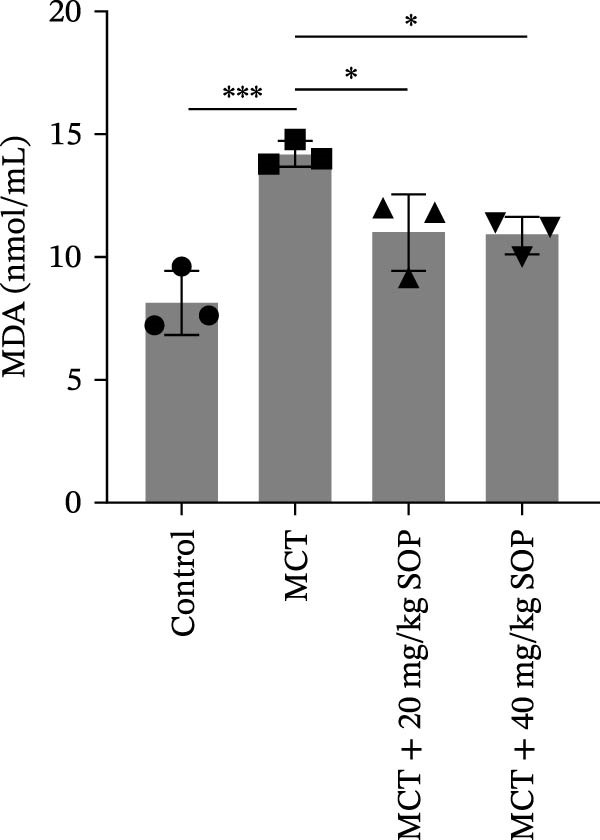
(D)

**Figure:**
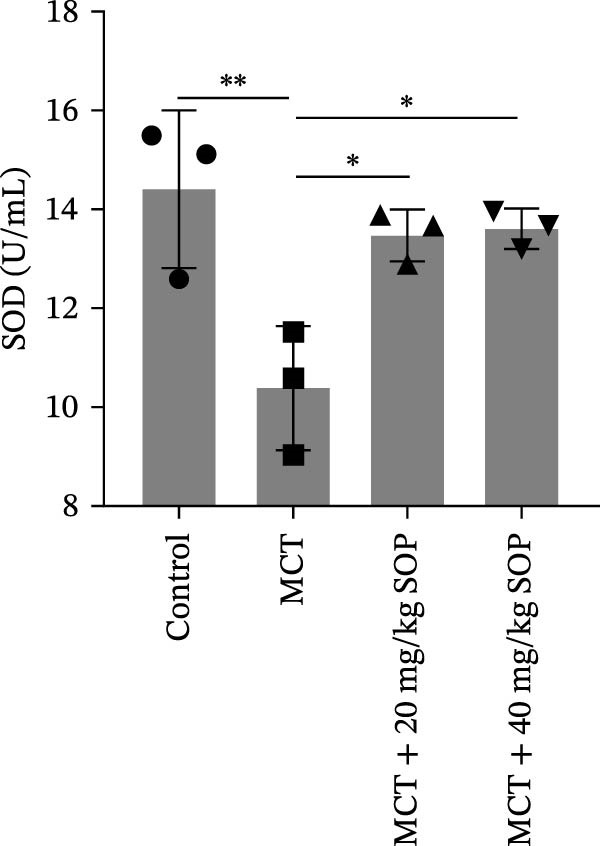
(E)

### 4.3. SOP Ameliorates MCT‐Induced Structural and Functional Changes in the Heart of PH Rats

We assessed the impact of SOP on RV structure and function in a rat model of PH induced by MCT. The intra‐observer coefficient of variation for echocardiographic data was <4%, and the inter‐observer correlation coefficient was >0.93, confirming the reproducibility of our color Doppler ultrasound findings. The results of our echocardiographic and right heart catheterization studies are detailed in Tables 1 and 2, and Figure 3. Our findings demonstrated a significant elevation in mPAP (Figure 3D) and RVSP in the MCT‐treated rats compared to controls, fulfilling the diagnostic criteria for PH (Figure 3H,I). Administration of SOP notably attenuated both pulmonary artery and RV pressures in the treated animals. The Fulton index was significantly increased in the MCT group relative to controls, indicating the progression of PH and concomitant hypertrophic remodeling of the right ventricle (Figure 3J). Conversely, SOP treatment substantially mitigated RV remodeling in the MCT‐induced PH rats. As shown in Table 2 and Figure 3, TAPSE and RVFAC were significantly diminished, while RVFWT was markedly elevated in the MCT group compared to controls. SOP treatment effectively reversed these alterations in a statistically significant manner (Figure 3A–C, E–G).

Figure 3Echocardiographic and right heart catheterization results in rats. (A–C), (E–G) Echocardiographic Detection of Cardiac Structural Alterations in Groups of Rats. (D) Mean pulmonary artery pressure waveforms of experimental rats in each group. (G–I) Right heart catheterization was used to detect RVSP and mPAP in all groups of rats. (J) Comparison of RV hypertrophy index in rats. Data are expressed as mean ± sd, *n* = 3 per group.

**Figure:**
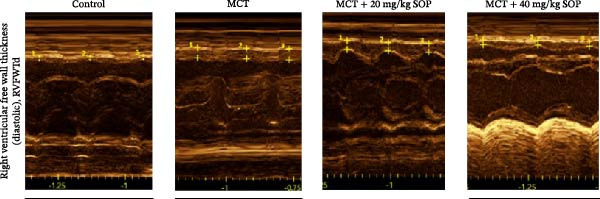
(A)

**Figure:**
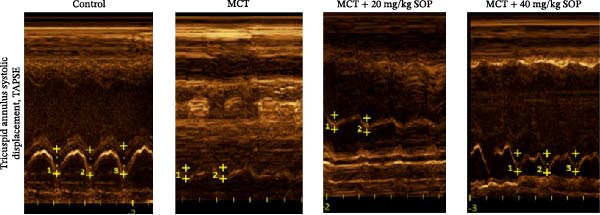
(B)

**Figure:**
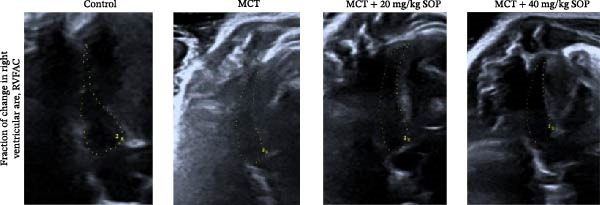
(C)

**Figure:**
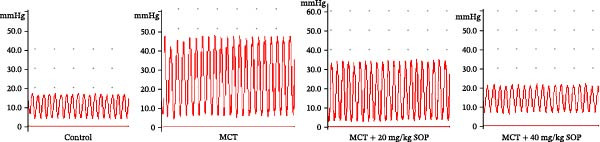
(D)

**Figure:**
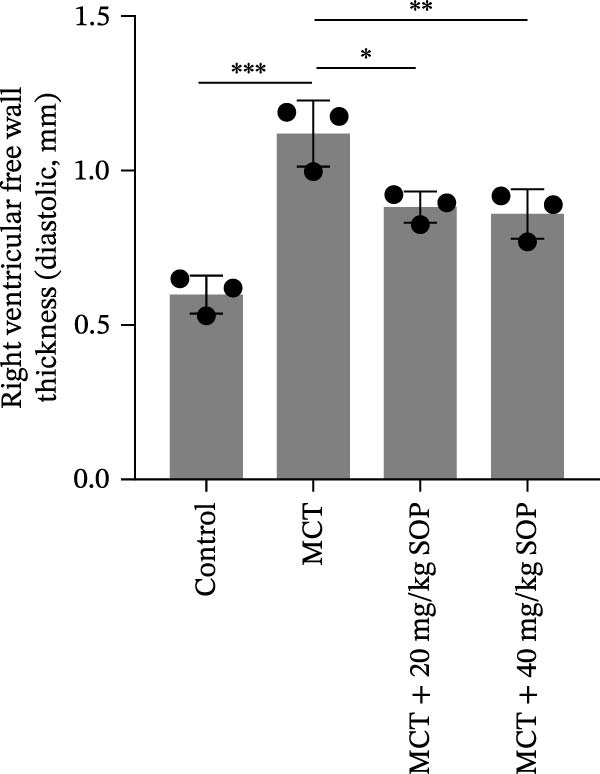
(E)

**Figure:**
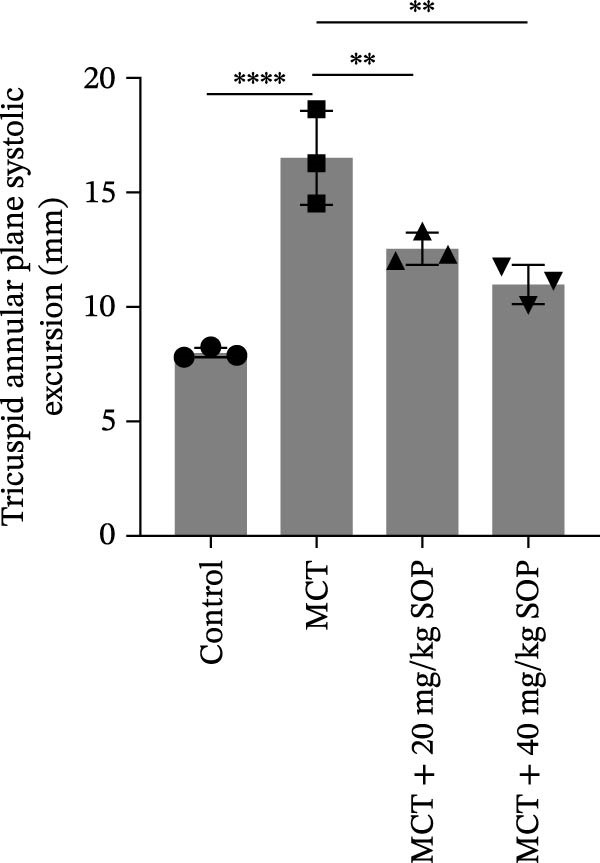
(F)

**Figure:**
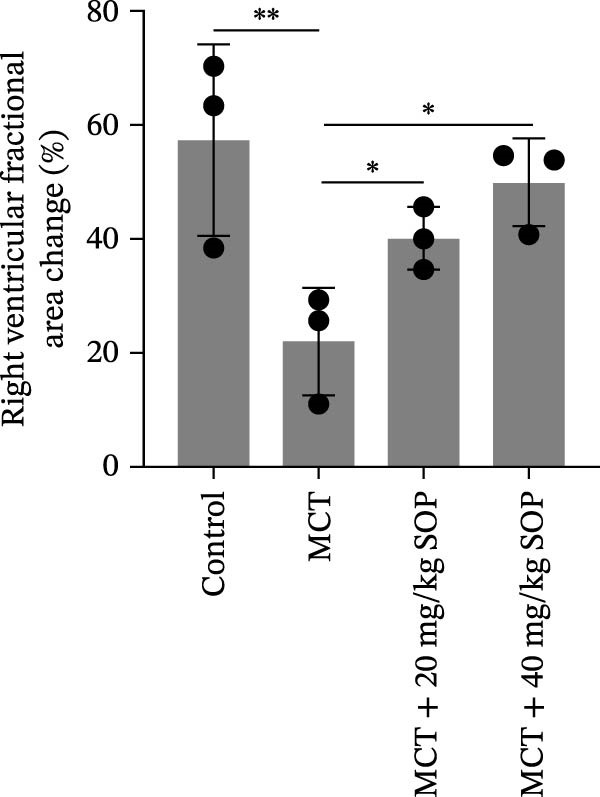
(G)

**Figure:**
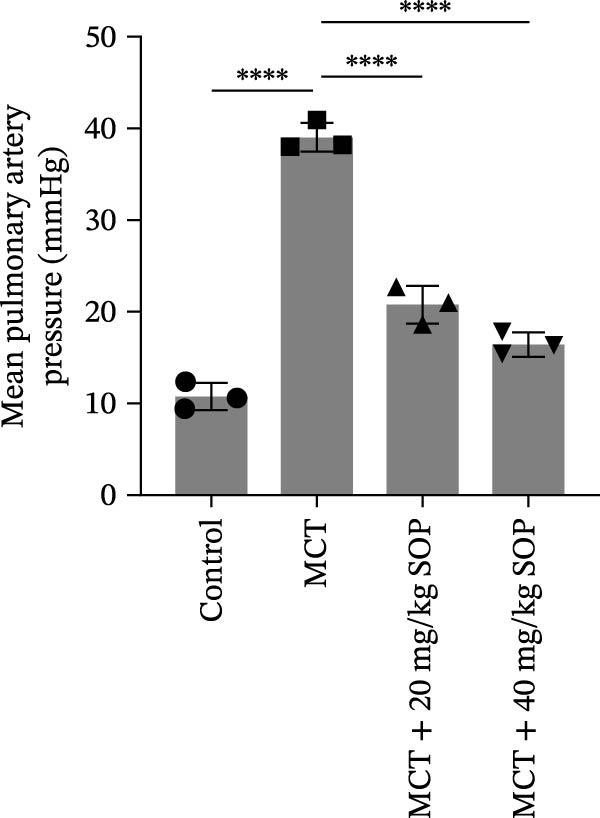
(H)

**Figure:**
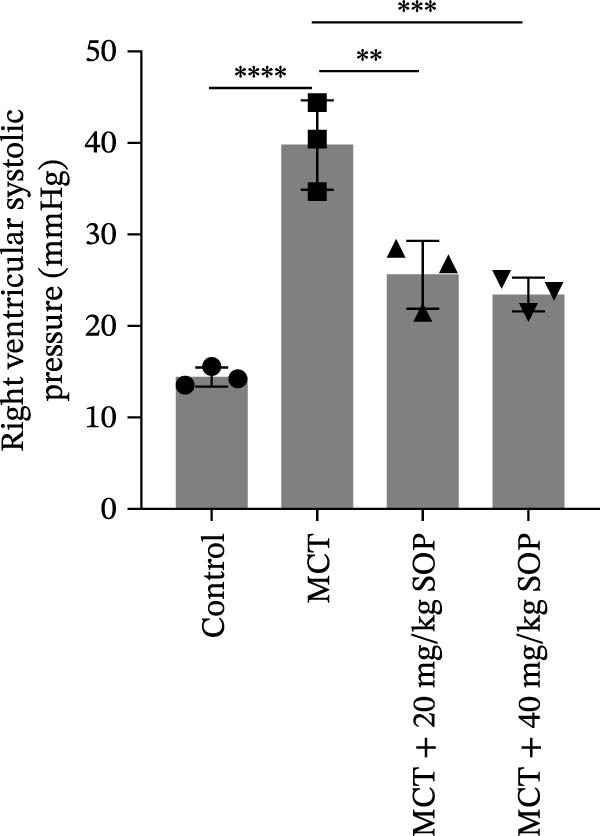
(I)

**Figure:**
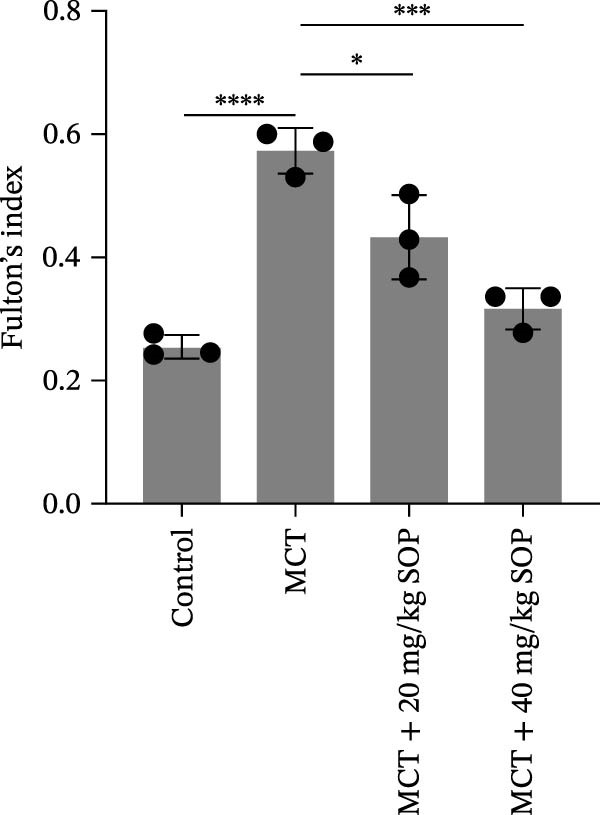
(J)

**Table 1:** Changes in mPAP, RVSP, and Fulton index in rats of all groups.

Group	mPAP, mmHg (mean ± sd, *p*)	RVSP, mmHg (mean ± sd, *p*)	Fulton’s index (mean ± sd, *p*)
Control	10.82 ± 1.50	14.42 ± 1.01	25.44 ± 1.87
MCT	39.13 ± 1.59 (<0.0001)	39.84 ± 4.87 (<0.0001)	57.22 ± 3.75 (<0.0001)
MCT + 20 mg/kg SOP	20.82 ± 2.06 (<0.0001)	25.60 ± 3.72 (0.0017)	43.25 ± 6.79 (0.0109)
MCT + 40 mg/kg SOP	16.45 ± 1.33 (<0.0001)	23.45 ± 1.90 (0.0007)	31.54 ± 3.40 (0.0002)

*Note:* The comparisons are between the MCT group and the Control group, and between the MCT + SOP group and the MCT group.

**Table 2:** Changes in ultrasound related indices in rats in each group.

Group	RVFWT (diastolic), mm (mean ± sd, *p*)	TAPSE, mm (mean ± sd, *p*)	RVFAC, % (mean ± sd, *p*)
Control	0.71 ± 0.09	3.01 ± 0.50	57.44 ± 16.81
MCT	1.23 ± 0.13 (0.0001)	1.49 ± 0.17 (<0.0001)	22.04 ± 9.61 (0.0098)
MCT + 20 mg/kg SOP	0.82 ± 0.13 (0.014)	1.96 ± 0.33 (0.08)	40.20 ± 5.53 (0.044)
MCT + 40 mg/kg SOP	0.70 ± 0.13 (0.0085)	2.23 ± 0.14 (0.0011)	49.95 ± 7.73 (0.033)

*Note:* The comparisons are between the MCT group and the Control group, and between the MCT + SOP group and the MCT group.

### 4.4. SOP Attenuates MCT‐Induced Pathological Changes in Lung and RV

We investigated the impact of SOP on the pathological alterations in the lungs and right ventricle in a rat model of MCT‐induced PH, utilizing both HE and Masson’s trichrome staining of tissue sections for assessment.

In the HE‐stained sections, the lung and RV cells of control rats appeared flattened, with pericytes organized into well‐defined vesicular structures (Figure 4A). The inner elastic lamina was intact and regular. The cytoplasm of the smooth muscle cells was densely populated with myofilaments and spindle‐shaped dense bodies, with distinct, prominent dense spots. In contrast, the MCT‐treated group exhibited thickened pulmonary artery walls, narrowed vessel lumens, and proliferative smooth muscle cells within the small pulmonary arteries. Endothelial hyperplasia and thrombus formation were also evident. RV cardiomyocytes displayed signs of proliferation and fibrosis, with increased smooth muscle cell size and organellar expansion. In the MCT + SOP groups, a phenotype intermediate between the control and MCT groups was observed, which suggests that SOP exerts a protective effect against the pathological changes in both the pulmonary and RV tissues. Notably, SOP administration was shown to attenuate RV hypertrophy and to slow the progression of cardiomyocyte proliferation and fibrosis, with the most significant effects observed in the high‐dose (40 mg/kg) group. Consistent with the Fulton index results, RV hypertrophy index, lumen/area ratio, and medial wall thickness also demonstrated the mitigating effect of SOP on cardiac remodeling in PH rats (Figure 4C–E).

Figure 4Results of HE staining of the pulmonary artery and Masson staining of the right ventricle in rats of each group. (A) HE staining results of lungs and right ventricles of rats in each group. (B) Results of Masson’s trichrome staining of the lungs and right ventricle of rats in each group. (C–E) Quantitative histological data on right ventricular wall thickness: histograms of right ventricular hypertrophy index, lumen/area ratio, and medial wall thickness. Data are expressed as mean ± sd, *n* = 3 per group.

**Figure:**
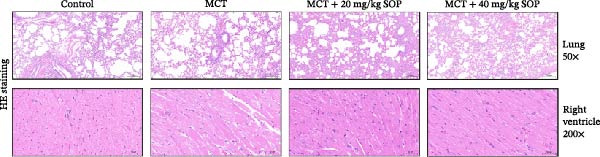
(A)

**Figure:**
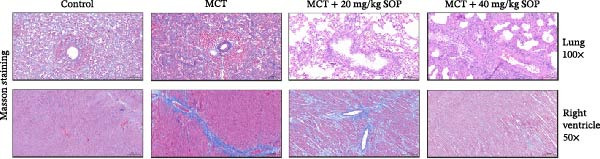
(B)

**Figure:**
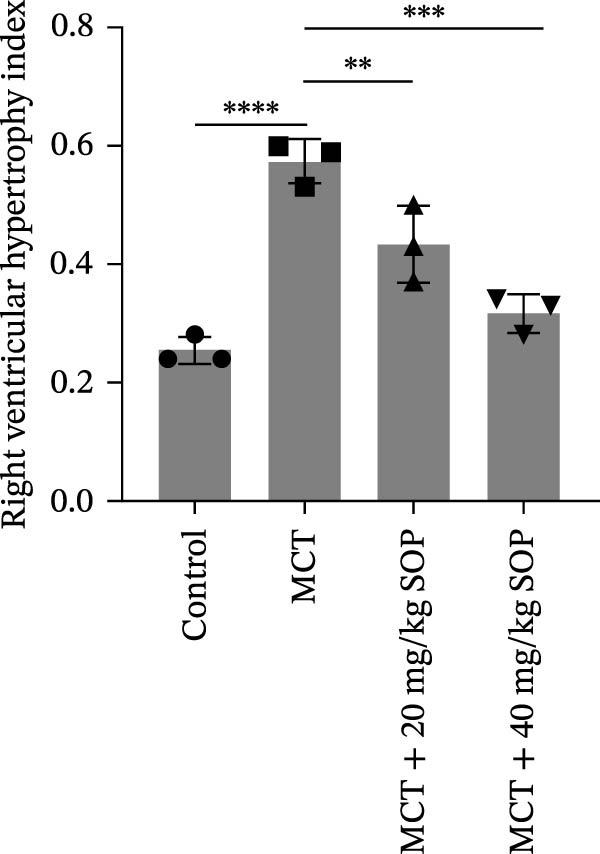
(C)

**Figure:**
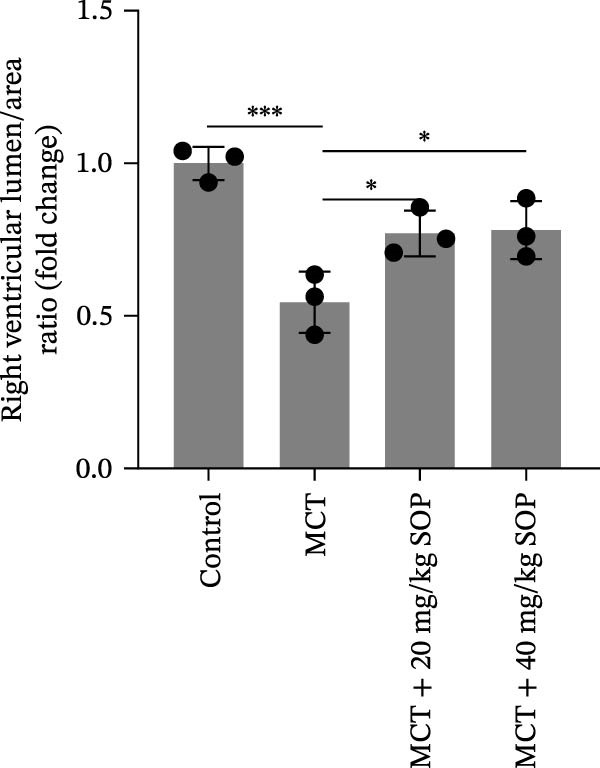
(D)

**Figure:**
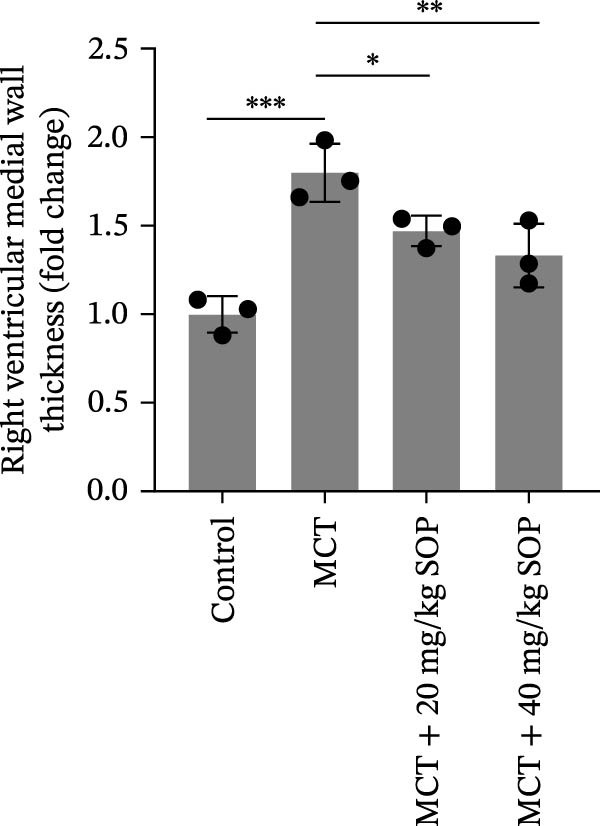
(E)

Additionally, fibrosis in lung and RV tissues was assessed via Masson’s trichrome staining (Figure 4B). Collagen deposition was significantly elevated in the lung tissues of the MCT group compared to the control, with fibrosis particularly pronounced in the interstitial spaces and surrounding small blood vessels. RV tissue demonstrated marked collagen accumulation, indicative of significant fibrotic changes and an expansion of the cardiomyocyte interstitial space. In contrast, the MCT + SOP group exhibited a notable reduction in collagen deposition within both lung and RV tissues, accompanied by a significant amelioration in the degree of fibrosis. These findings underscore the efficacy of SOP in mitigating structural damage to the heart. Furthermore, the MCT + 40 mg/kg SOP group demonstrated a more pronounced improvement compared to the MCT + 20 mg/kg SOP group.

### 4.5. SOP Ameliorates Inflammation, Apoptosis, and Vascular Remodeling in MCT‐Induced PH

We investigated the impact of SOP on inflammatory responses, apoptosis, and vascular remodeling in the MCT‐induced PH rat model and subsequently examined the expression of key proteins through Western blot analysis.

In this model, we assessed the levels of inflammation and oxidative stress in PH rats by quantifying the expression of pro‐inflammatory mediators (IL‐1β and TLR4) and oxidative stress markers (iNOS and SOD‐1) in lung tissues (Figure 5A,D–F,J). The results revealed a marked elevation in the expression of IL‐1β, TLR4, and iNOS in the lung tissues of the MCT‐treated rats, compared to controls. However, in the SOP‐treated group, a notable reduction in the expression of these inflammatory mediators was observed, suggesting that SOP may mitigate MCT‐induced inflammation and oxidative stress through the suppression of these factors’ upregulation.

Figure 5SOP ameliorates inflammation, apoptosis, and vascular remodeling in the PH model. (A–K) The protein expression levels of fibrosis‐related proteins such as NRF2, HO‐1, TLR4, IL‐1β, iNOS, vimentin, Bcl‐2, α‐SMA, SOD‐1, and Bax were detected using a Western blotting assay. (L–P) Flow cytometry to detect apoptosis in each group of cells. Data are expressed as mean ± sd, *n* = 3 per group.

**Figure:**
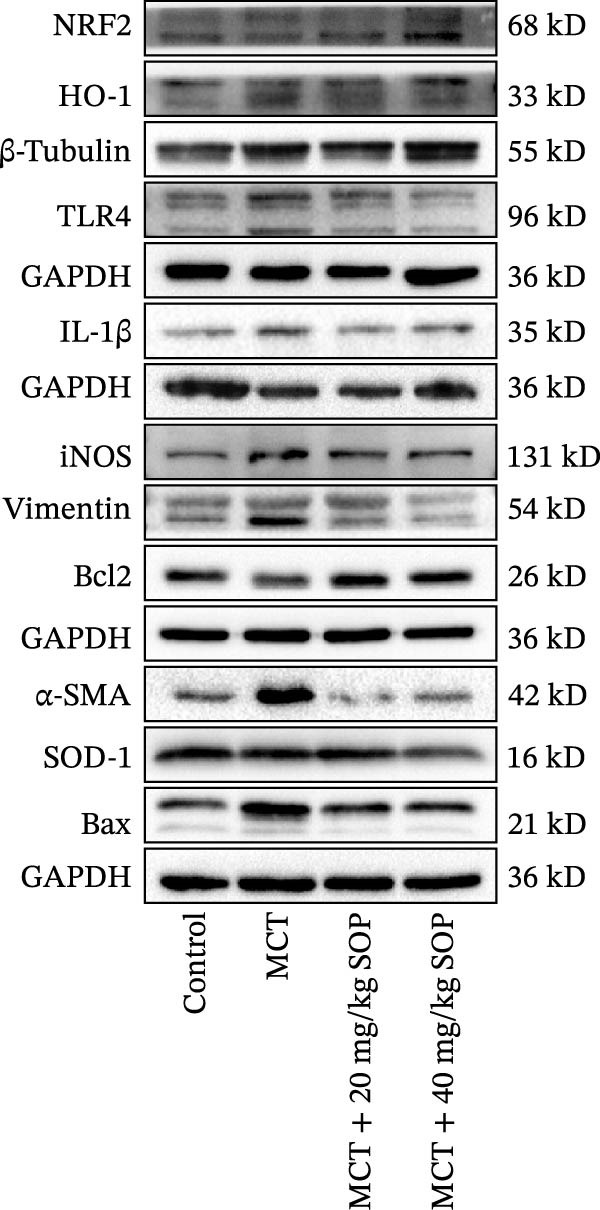
(A)

**Figure:**
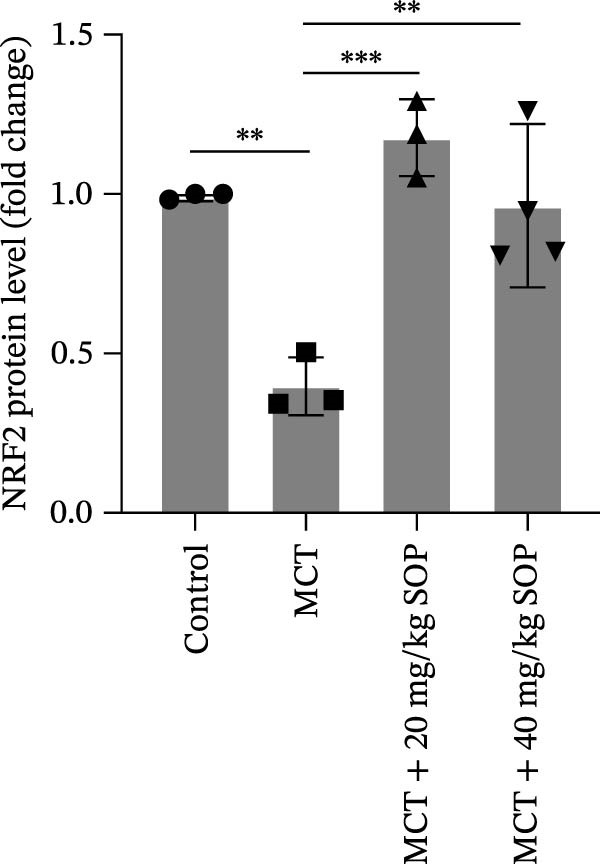
(B)

**Figure:**
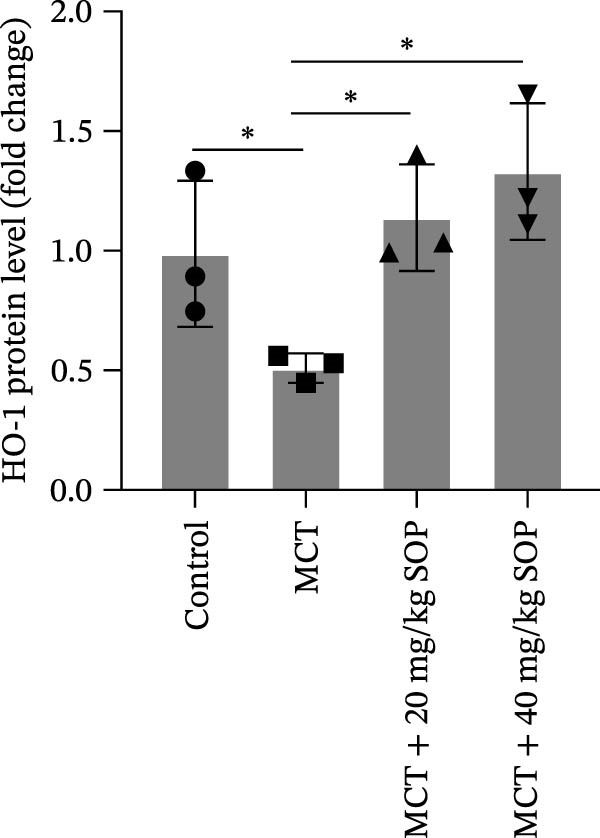
(C)

**Figure:**
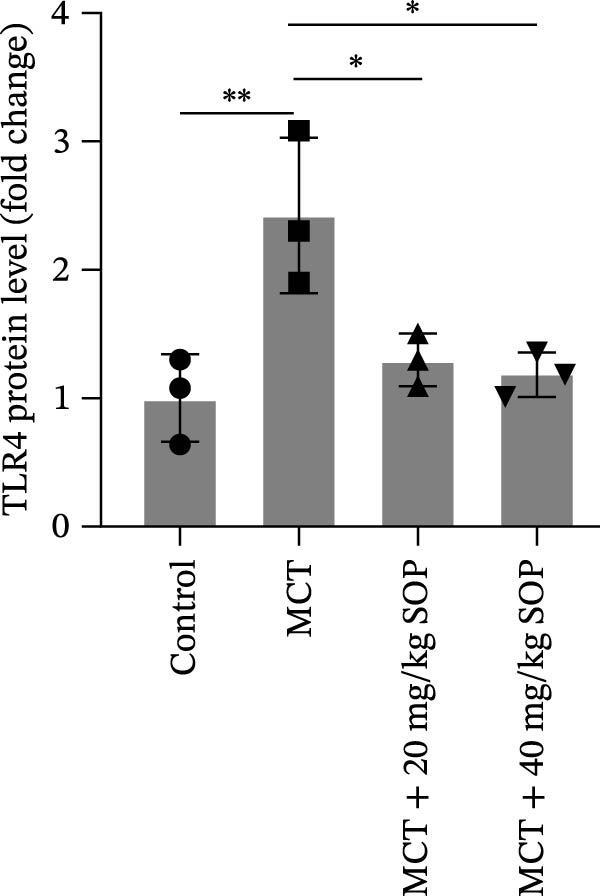
(D)

**Figure:**
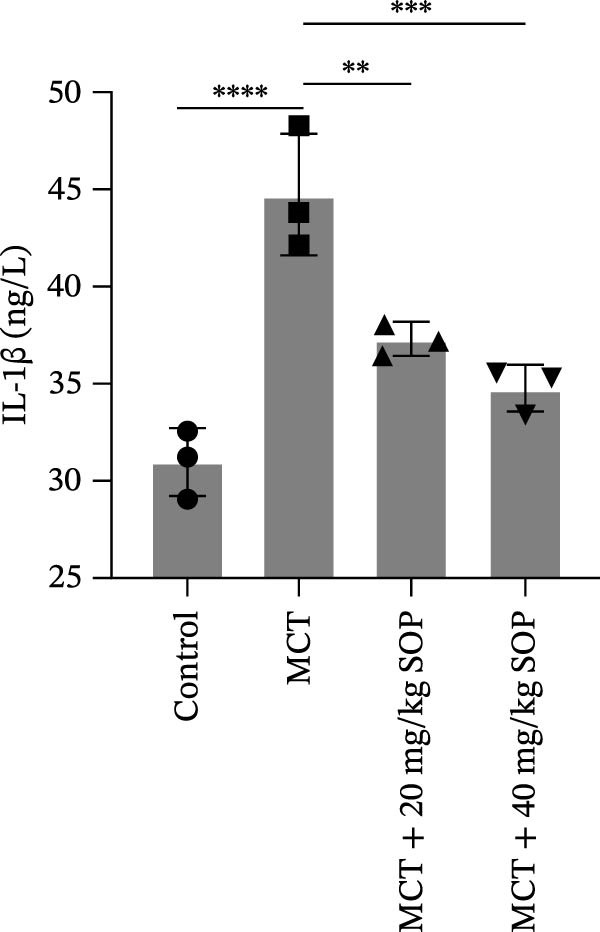
(E)

**Figure:**
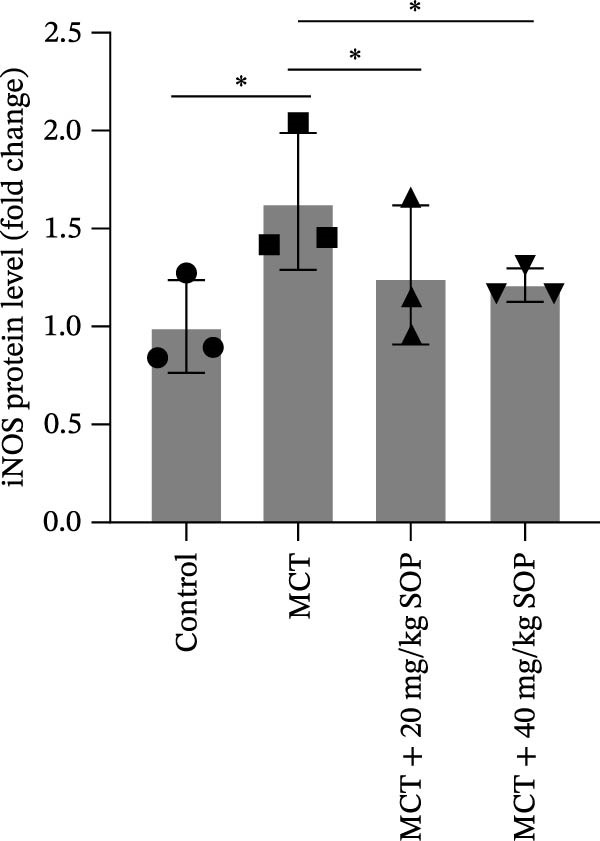
(F)

**Figure:**
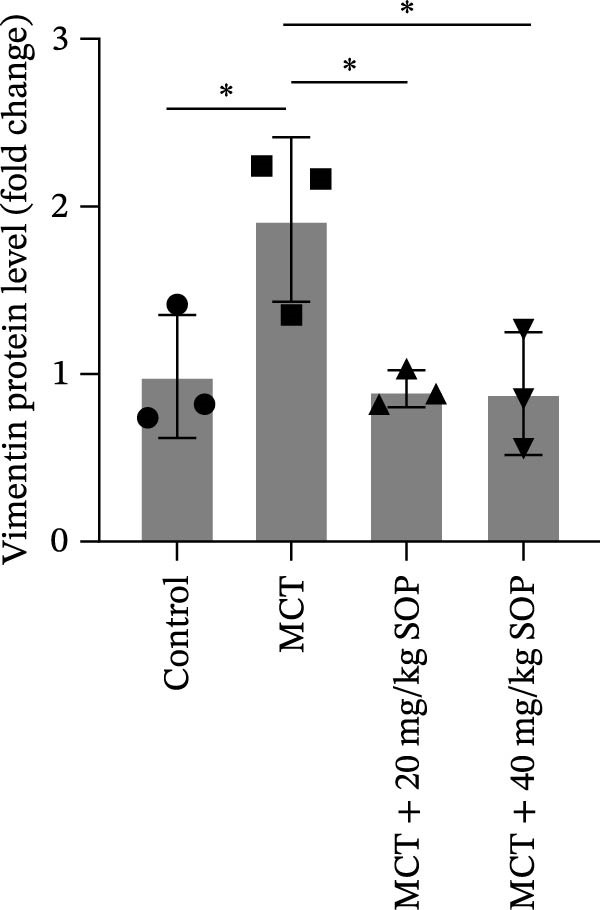
(G)

**Figure:**
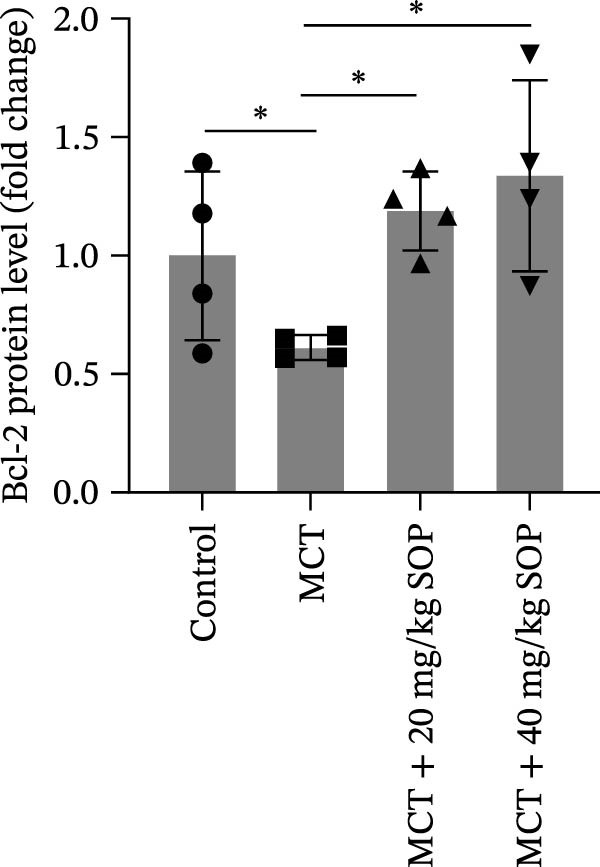
(H)

**Figure:**
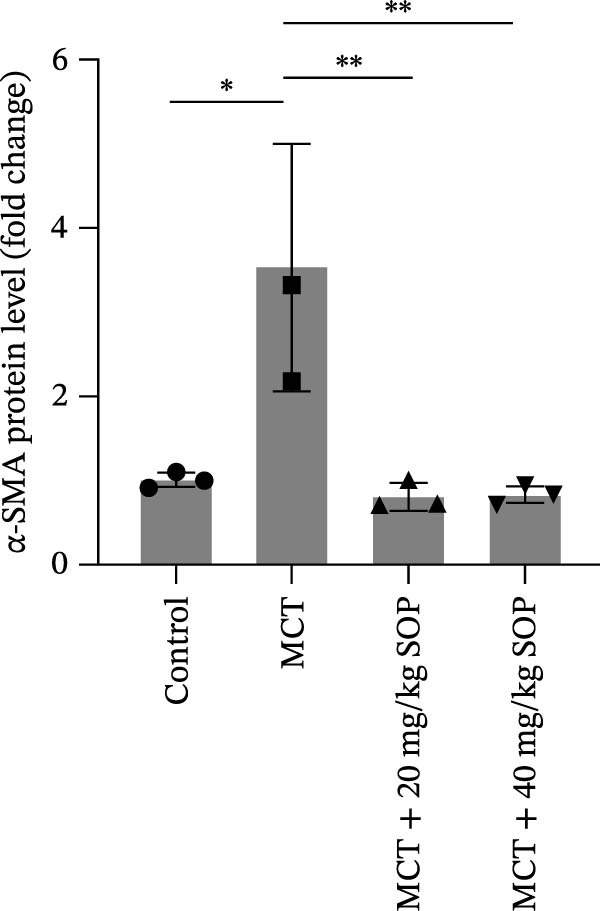
(I)

**Figure:**
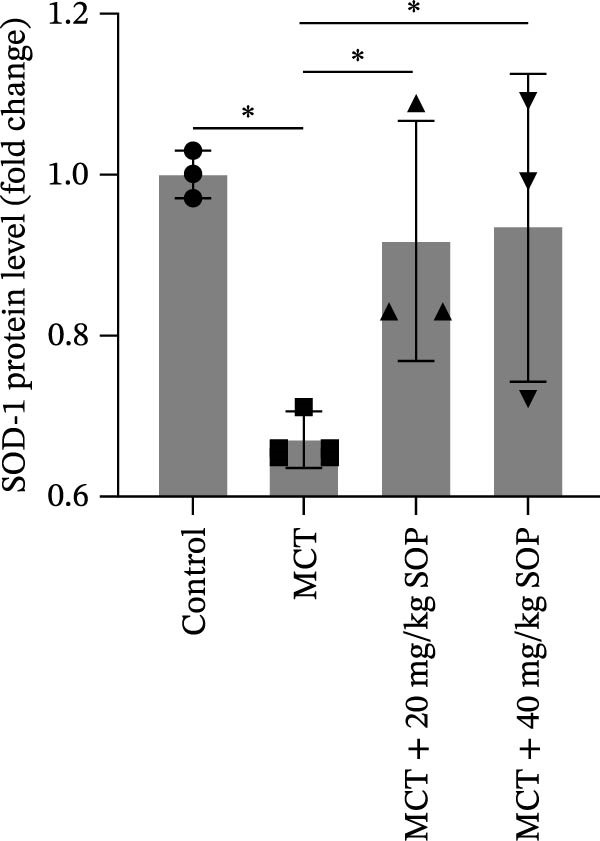
(J)

**Figure:**
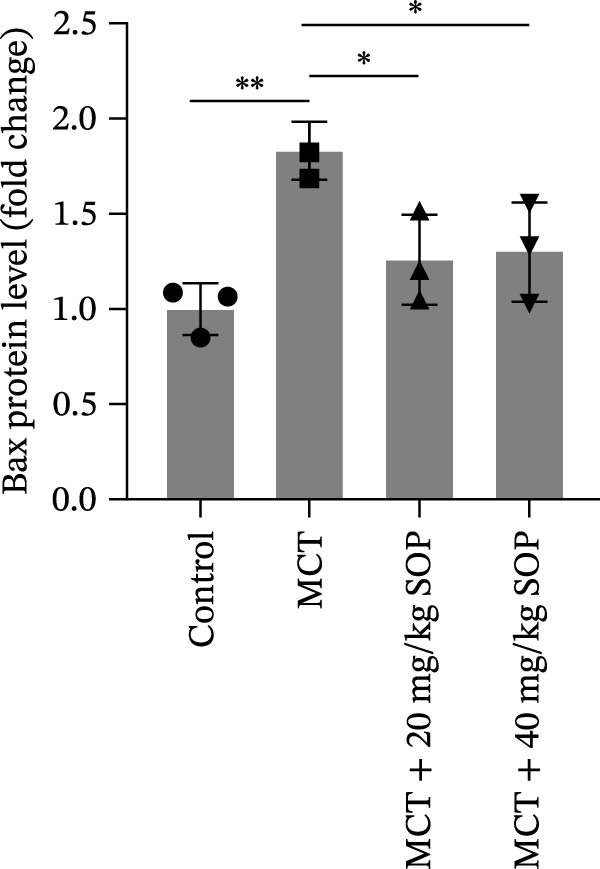
(K)

**Figure:**
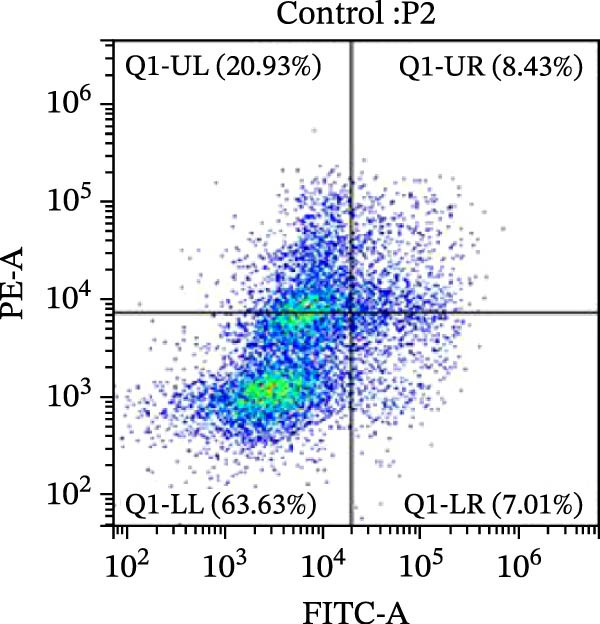
(L)

**Figure:**
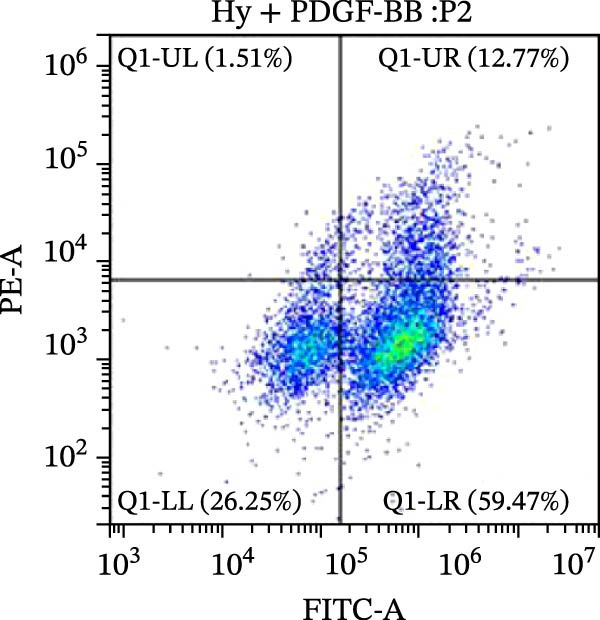
(M)

**Figure:**
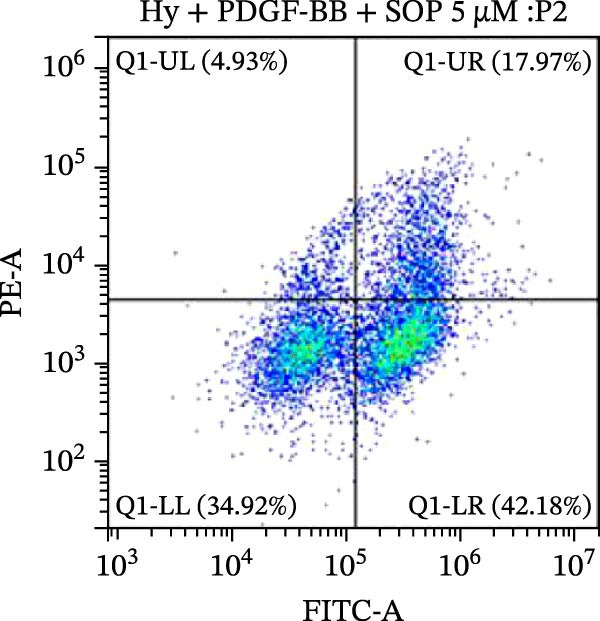
(N)

**Figure:**
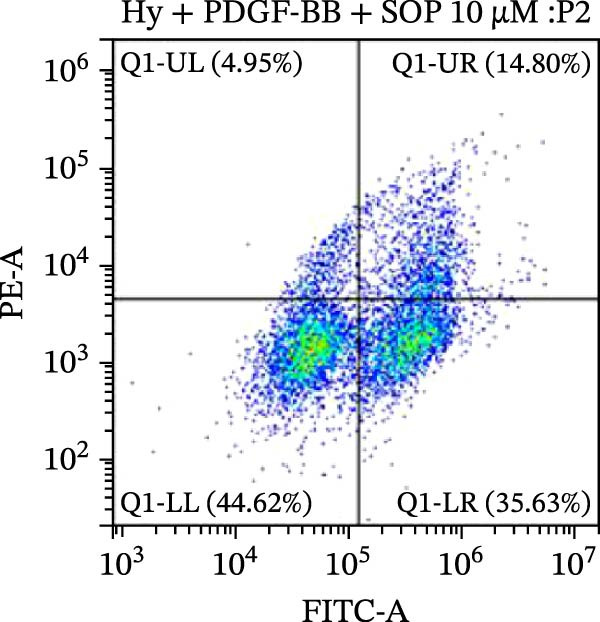
(O)

**Figure:**
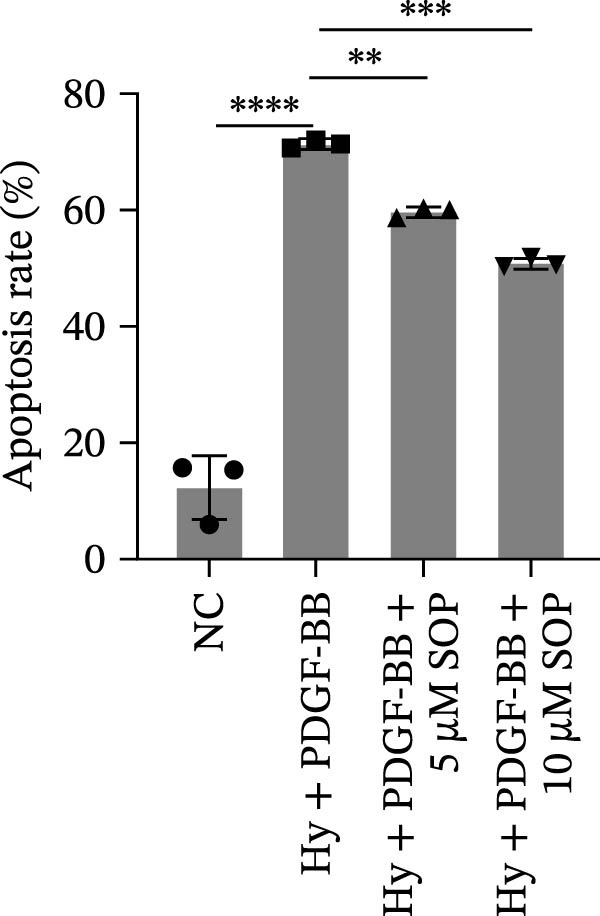
(P)

In terms of apoptosis, the expression of the pro‐apoptotic protein Bax was markedly upregulated, while the anti‐apoptotic protein Bcl‐2 was significantly downregulated in the MCT group compared to the control group, resulting in an elevated Bax/Bcl‐2 ratio. This shift in balance likely contributes to an increased rate of apoptosis (Figure 5H,K). Notably, the Bax/Bcl‐2 ratio was substantially reduced in the SOP‐treated group, suggesting that SOP effectively mitigates cell apoptosis. Meanwhile, we also used flow cytometry to analyze the apoptosis rate, and the results are shown in Figure 5M–Q. Compared with the control group, the PH model induced aberrant apoptosis in HPASMCs.

Regarding vascular remodeling, Western blot analysis revealed a significant upregulation of pulmonary artery smooth muscle cell markers α‐SMA and vimentin in the MCT group relative to controls, indicating pronounced vascular smooth muscle cell proliferation and activation (Figure 5G,I). In contrast, SOP treatment led to a significant decrease in the expression of α‐SMA and vimentin, implying that SOP effectively curtails the proliferation of vascular smooth muscle cells, thereby inhibiting both vascular and myocardial remodeling.

In HPASMCs, consistently, Western blot analysis showed results consistent with those observed in animal experiments (Figure 6A–L).

Figure 6SOP mitigates PH progression by activating NRF2‐dependent antioxidant, anti‐inflammatory, and antifibrotic pathways. (A–L) The protein expression levels of fibrosis‐related proteins such as cleaved caspase‐3, NRF2, HO‐1, TLR4, IL‐1β, iNOS, vimentin, Bcl‐2, α‐SMA, SOD‐1, and Bax were detected using a Western blotting assay. (M–Q) By the ML385 blocking assay, it is clear that the improvement of the PH cell phenotype by SOP is dependent on the activation of the NRF2/HO‐1 pathway. Data are expressed as mean ± sd, *n* = 3 per group.

**Figure:**
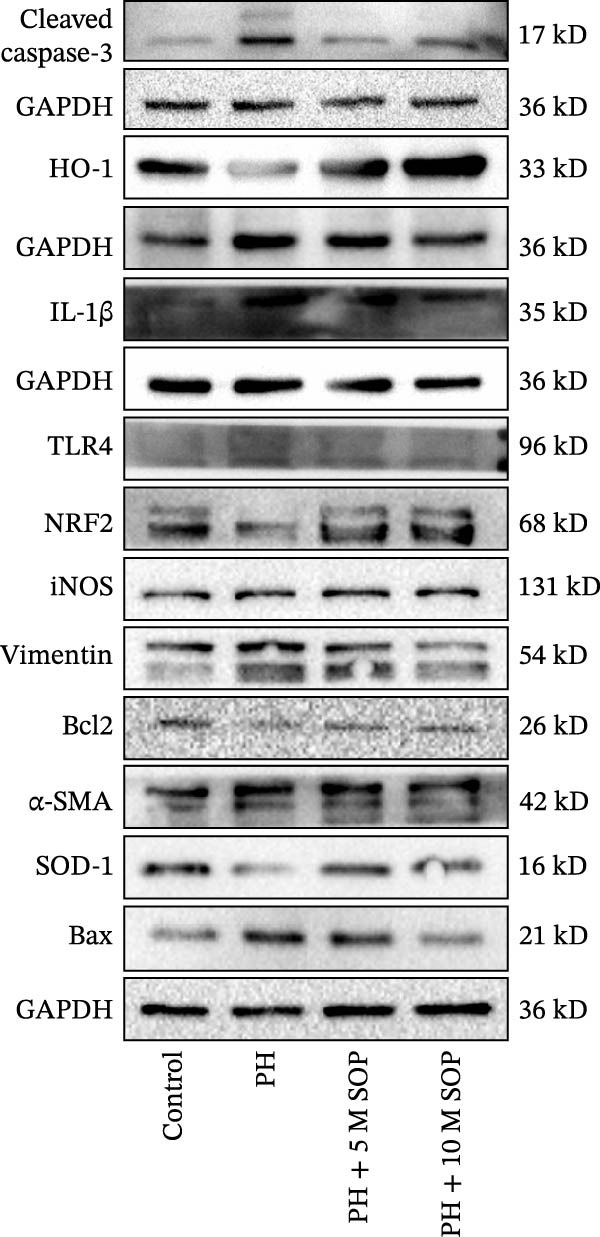
(A)

**Figure:**
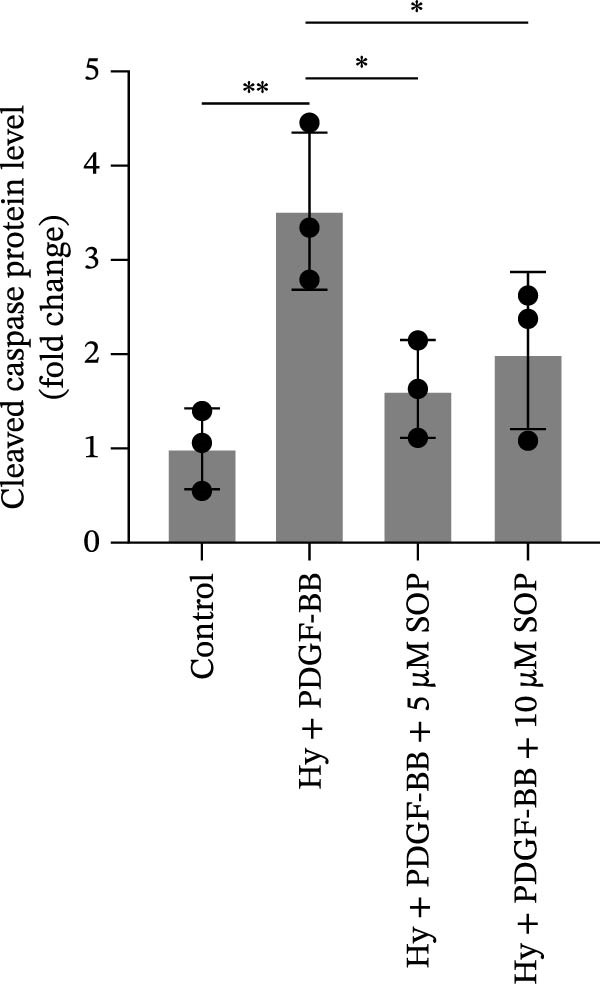
(B)

**Figure:**
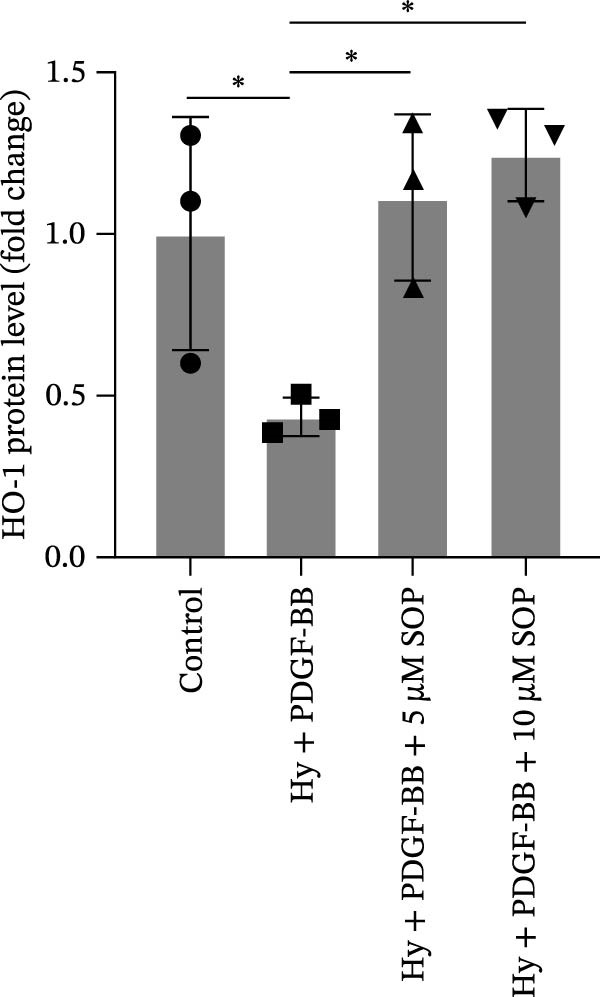
(C)

**Figure:**
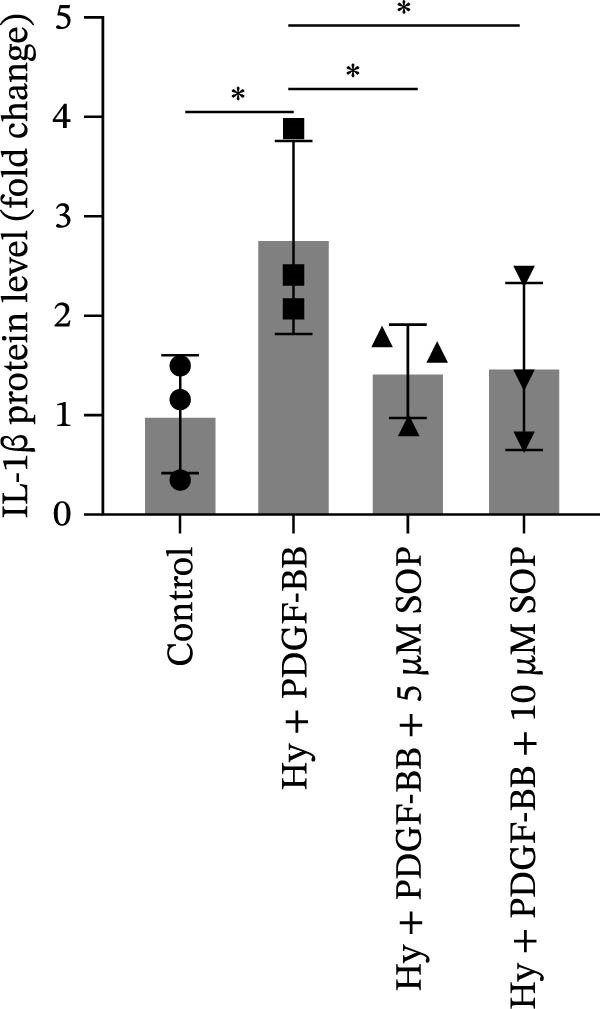
(D)

**Figure:**
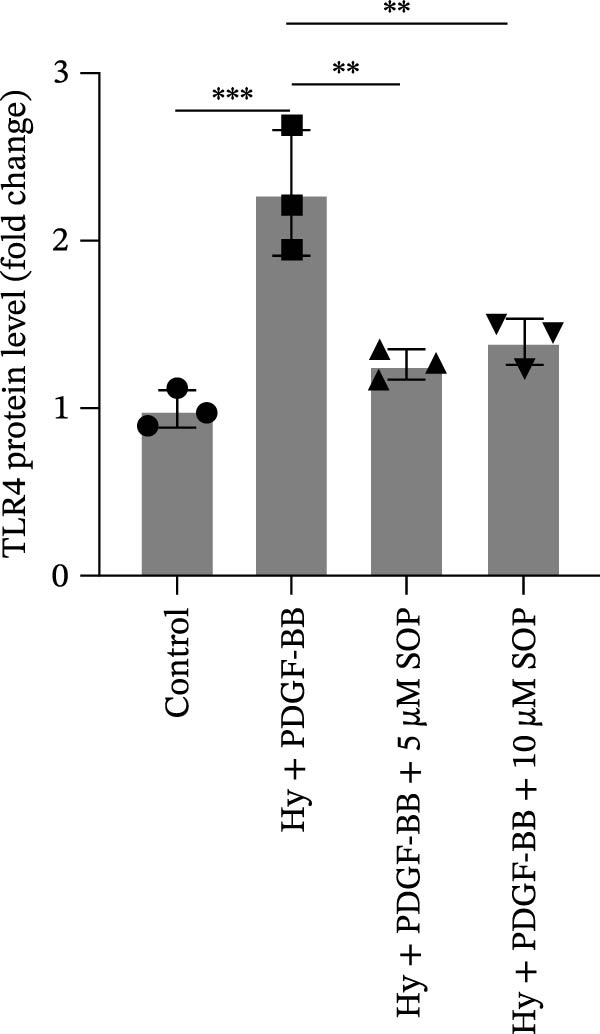
(E)

**Figure:**
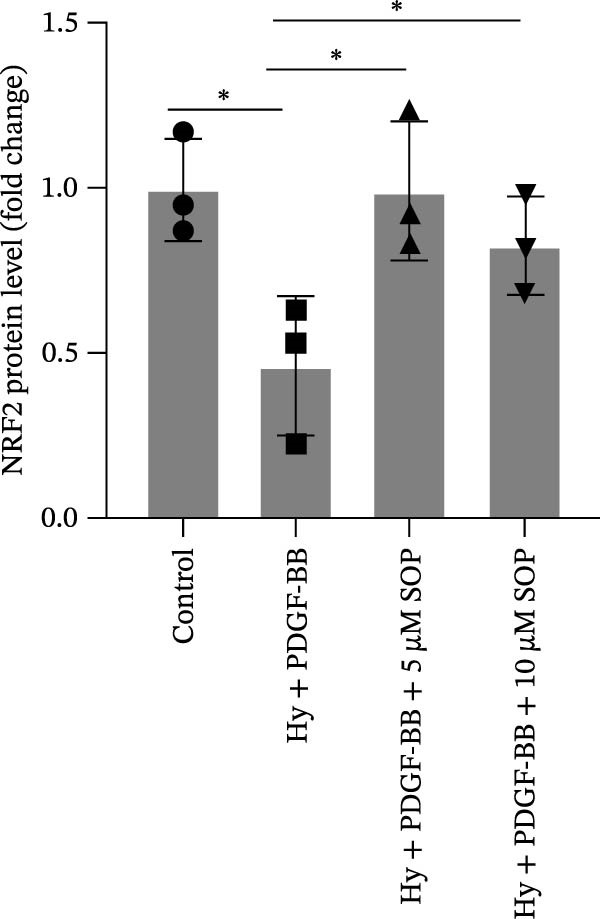
(F)

**Figure:**
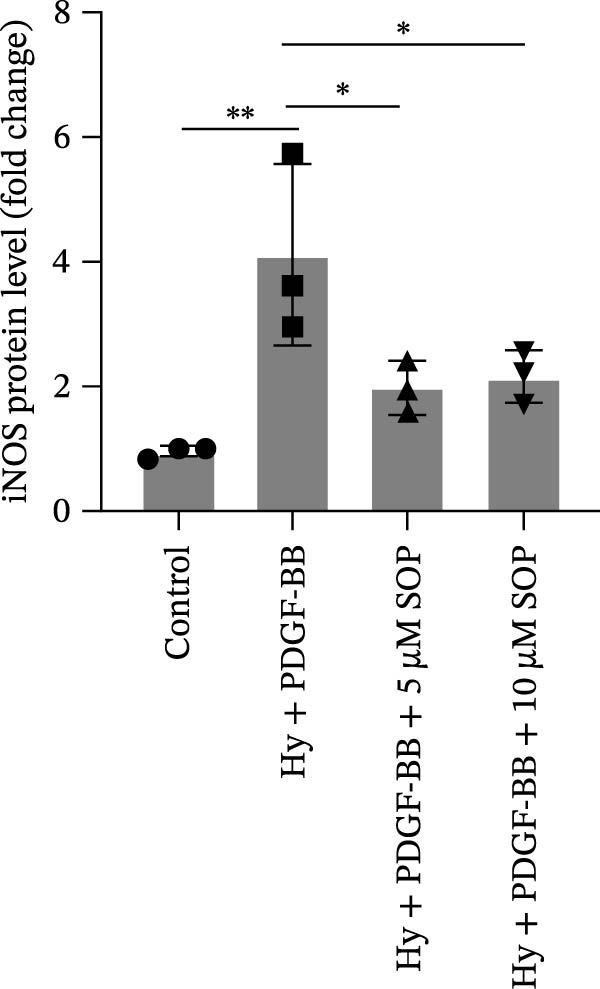
(G)

**Figure:**
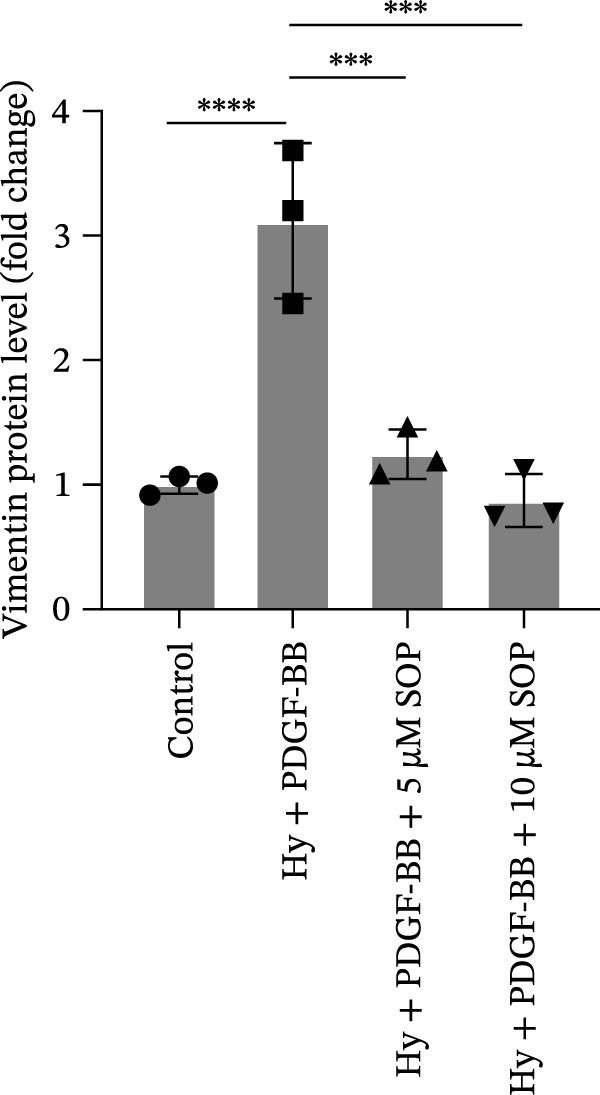
(H)

**Figure:**
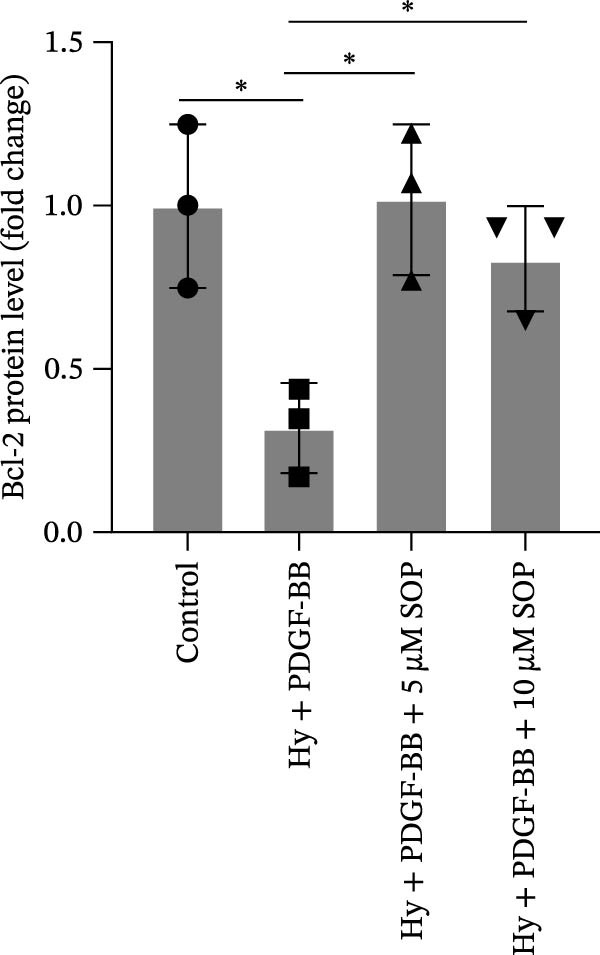
(I)

**Figure:**
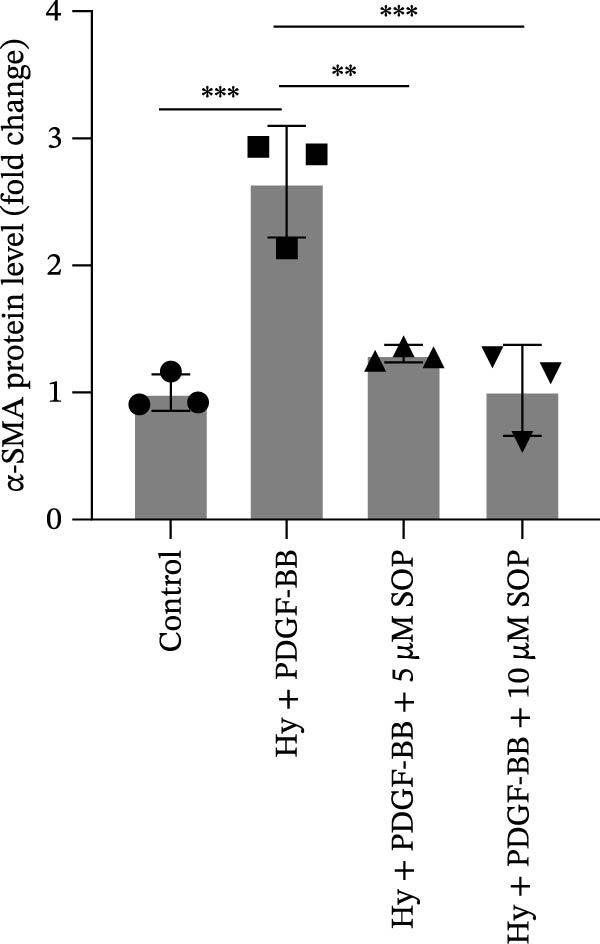
(J)

**Figure:**
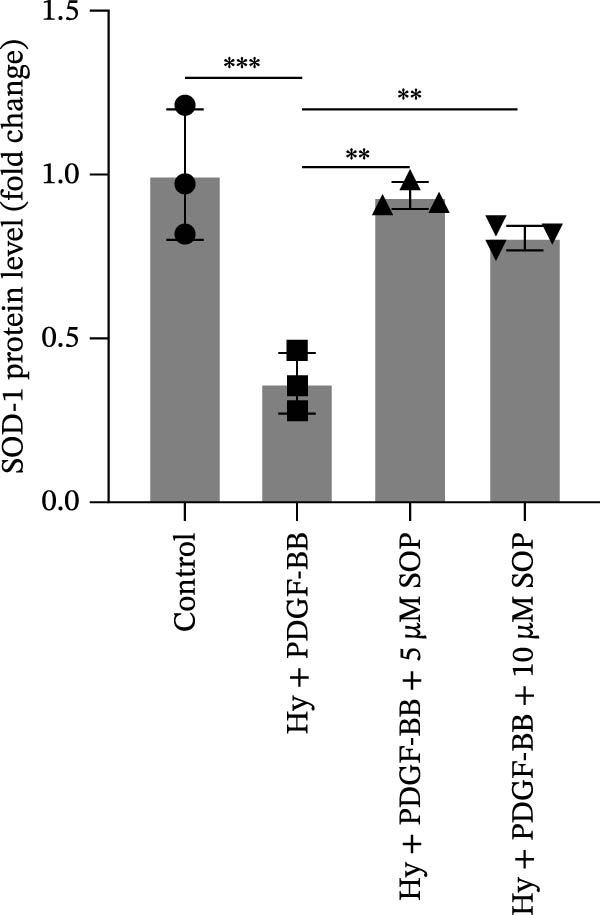
(K)

**Figure:**
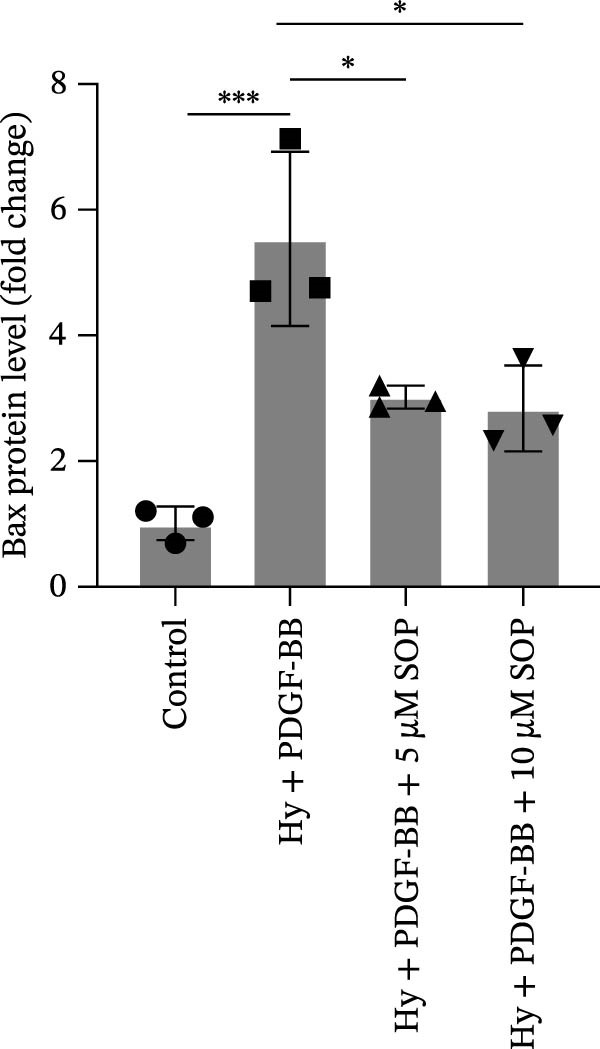
(L)

**Figure:**
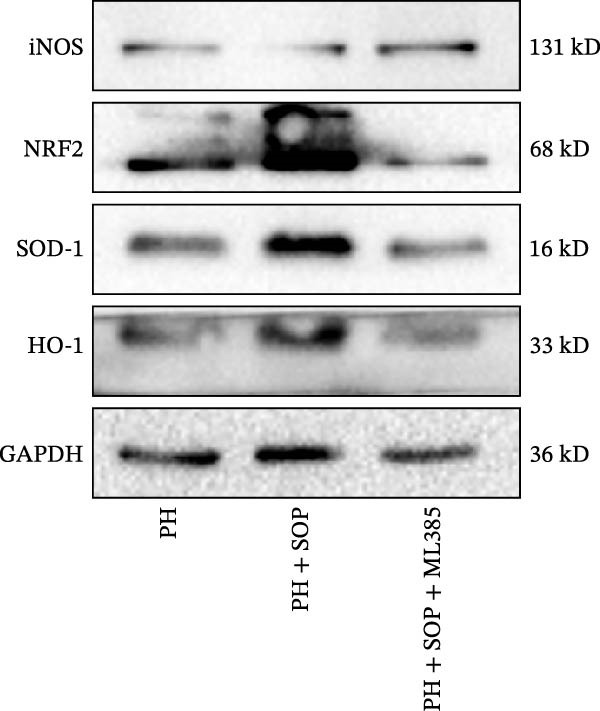
(M)

**Figure:**
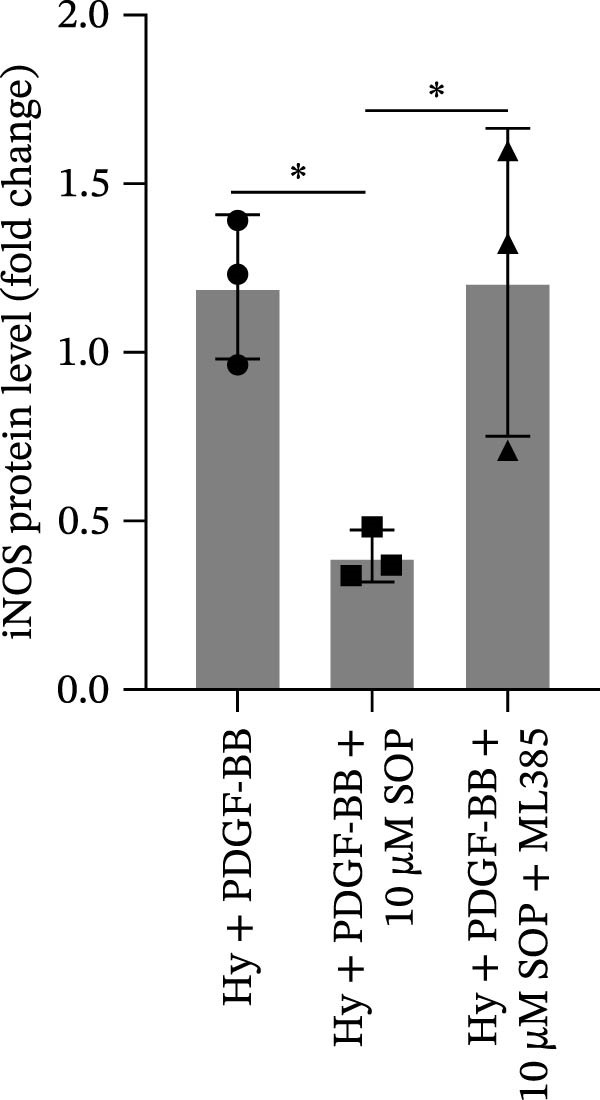
(N)

**Figure:**
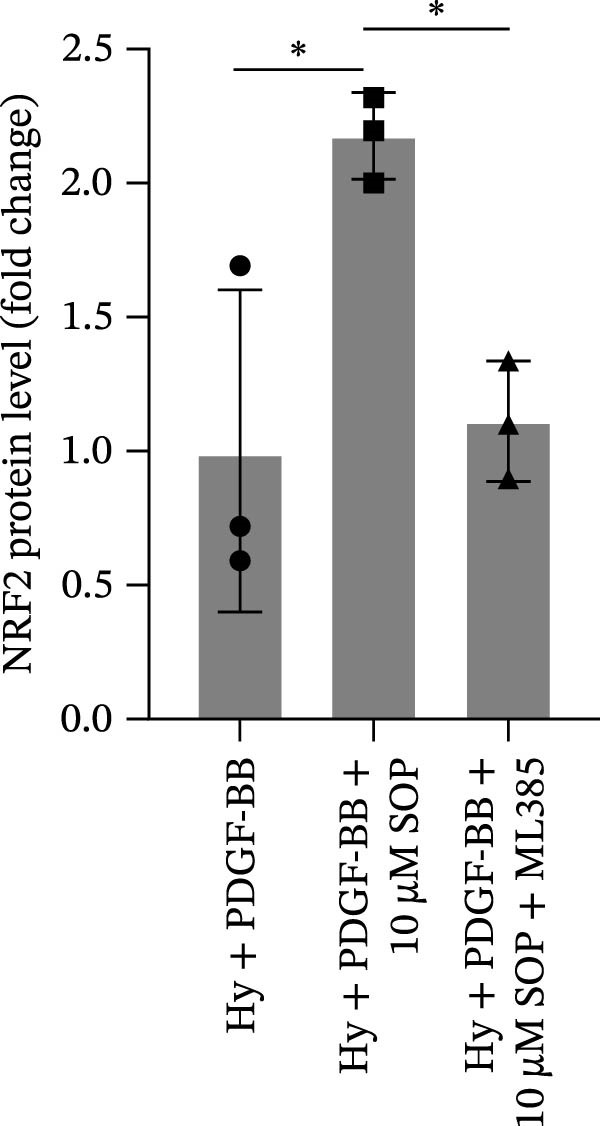
(O)

**Figure:**
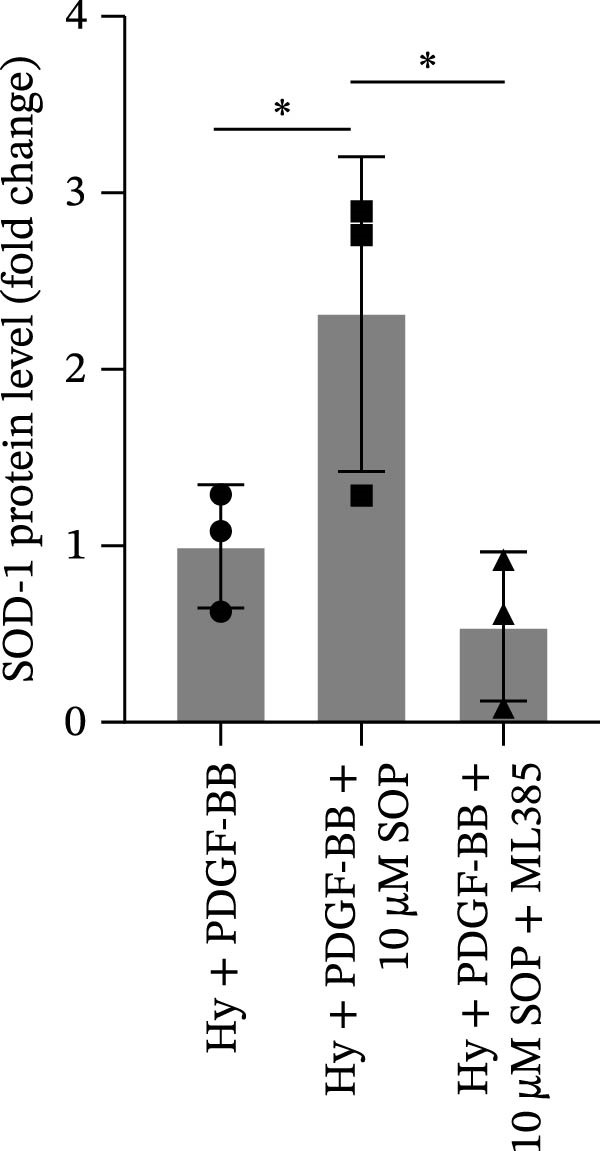
(P)

**Figure:**
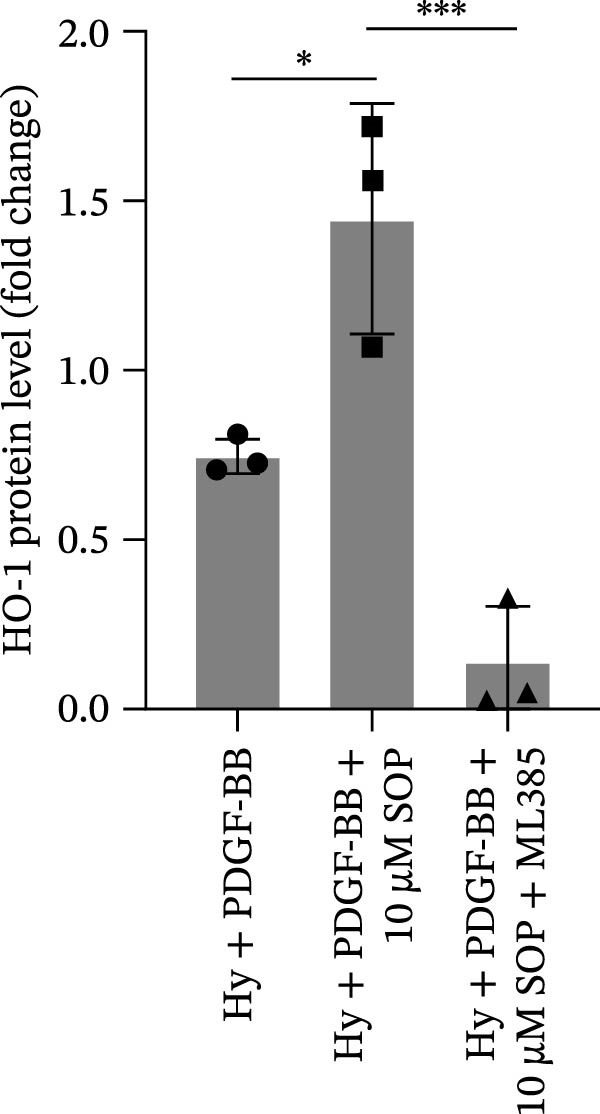
(Q)

### 4.6. SOP Exerts Its Effects Potentially Through Activation of NRF2/HO‐1

**Figure 7:**
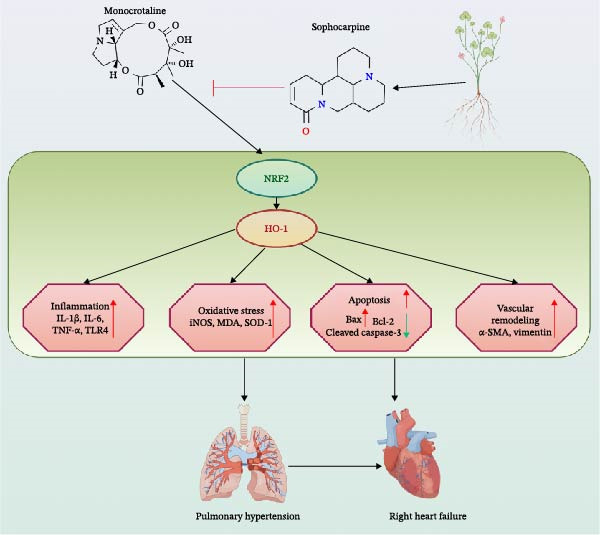
Possible mechanisms by which SOP ameliorates MCT‐induced injury in PH rats. SOP attenuates MCT‐induced PH by inhibiting inflammation, apoptosis, oxidative stress, and fibrosis, consistent with activation of the NRF2/HO‐1 signaling pathway.

To further elucidate the potential mechanisms underlying the beneficial effects of SOP on PH, we investigated the *in vivo* expression of the NRF2/HO‐1 signaling pathway in PH rats and HPASMCs. Our findings indicated that MCT suppressed the NRF2/HO‐1 pathway, whereas SOP treatment activated it (Figures 5B,C and 6C,F). We then used ML385, an inhibitor of the NRF2/HO‐1 pathway, to divide the cells into three groups and verify the mechanistic pathway through which SOP acted (Figure 6M–Q). We found that SOP inhibited the abnormal HP‐phenotype (iNOS) and enhanced SOD‐1 in HPASMCs by activating the NRF2/HO‐1 pathway. Blocking this pathway, however, counteracted the protective effect of SOP. SOP protection was counteracted by blocking this pathway. These findings indicate that SOP alleviates MCT‐induced pathological changes, including inflammation, oxidative stress, fibrosis, and RV remodeling, potentially through activation of the NRF2/HO‐1 pathway (Figure 7).

## 5. Discussion

Current treatment for PH primarily focuses on alleviating symptoms and acutely reducing pulmonary artery pressure [19]. However, long‐term regulation of pulmonary vascular remodeling is often‐overlooked, and this unmet need limits therapeutic efficacy [20]. Here, we report two key novel insights into SOP’s protective effects in PH, addressing gaps in current understanding and therapy.

First, as a multi‐targeted drug, SOP simultaneously alleviates the four interrelated pathological features of PH: oxidative stress, inflammation, abnormal apoptosis, and vascular/ventricular remodeling [21–24]. Unlike single‐mechanism therapies, this broad‐spectrum activity aligns with the complex pathophysiological mechanisms of PH, where these processes synergistically drive disease progression [25–27]. Second, SOP may mediate these effects by activating the NRF2/HO‐1 pathway, linking antioxidant defense with immune regulation. Notably, SOP reduces macrophage and T‐cell infiltration while suppressing pro‐inflammatory cytokine release (IL‐1β, TNF‐α), directly addressing the often‐overlooked immune dysregulation in PH [28, 29]. The SOP reduces reactive oxygen species accumulation and lipid peroxidation by enhancing superoxide dismutase activity and lowering MDA levels. This, in turn, accelerates endothelial dysfunction, vascular remodeling, and pathological alterations within the pulmonary vasculature [30, 31]. Moreover, TLR4—a toll‐like receptor family pattern recognition receptor—is widely expressed on immune cells, endothelial cells, and smooth muscle cells [32]. It triggers immune responses by recognizing pathogen‐associated molecules or host‐derived damage‐associated molecular patterns (DAMPs), activating downstream signaling to start inflammatory cascades that mediate both pathogen defense and endogenous injury detection. In PH, key inflammatory mediators drive the pathological process. Elevated TNF‐α promotes HPASMC proliferation, pulmonary arterial wall remodeling, and collagen deposition, exacerbating vascular structural changes [33]. Increased IL‐1β impairs pulmonary endothelial function, intensifies inflammatory responses, and induces vasoconstriction [34]. Collectively, these effects worsen the clinical manifestations of PH. Notably, recent studies indicate that anti‐inflammatory drugs (such as IL‐1 receptor antagonists) can improve pulmonary artery pressure and function in PH models, supporting targeted anti‐inflammatory therapies as a promising treatment strategy [35, 36].

PH pathophysiology also hinges on abnormal HPASMC proliferation and apoptosis. Endothelial or smooth muscle cell apoptosis disrupts vasodilatory function, prompting release of provasoconstrictive factors (e.g., endothelin‐1, platelet‐derived growth factor) that stimulate HPASMC proliferation and vascular thickening [37]. An imbalance between PASMC apoptosis and proliferation drives vessel wall thickening, increased pulmonary vascular resistance, and PH progression. RV muscle cell apoptosis further impairs cardiac function and induces fibrosis, worsening secondary right heart failure [38–40]. Vascular remodeling, which is the hallmark of PH, is defined by the proliferation of PASMC and the thickening of the intima. This process is amplified by inflammatory mediators such as IL‐1β, which promote proliferation, apoptosis, and cell migration.

Second, the SOP has been demonstrated to elicit antioxidant and anti‐inflammatory effects through the activation of the NRF2/HO‐1 pathway [41–43]. Conclusive evidence from prior studies indicates that NRF2/HO‐1 functions as a pivotal defense mechanism against oxidative stress associated with PH. The present study explores the relationship between vascular injury and the cross‐regulation with inflammatory signaling, a topic that has been rarely explored in the existing literature. This study further reveals that SOP acts as a novel activator of the NRF2/HO‐1 pathway, translating its protective effects into synergistic improvements in vascular structure, cardiac function, and immune homeostasis. The therapeutic effects of SOP are achieved through the restoration of redox balance, as evidenced by the enhancement of SOD activity and the reduction of MDA and ROS levels. Additionally, the modulation of apoptosis‐related protein expression, including Bax/Bcl‐2 and cleaved caspase‐3, contributes to the therapeutic response. This approach is predicated on the premise that it will address the downstream functional abnormalities of the pathway while concomitantly targeting upstream inflammatory triggers. The present study suggests that SOP may not only activate the NRF2/HO‐1 pathway (e.g., by promoting NRF2 nuclear translocation and HO‐1 upregulation to scavenge free radicals) but also amplify anti‐inflammatory effects. This synergistic mechanism is novel in that it links antioxidant defense with immune modulation, thereby ensuring that oxidative stress reduction is not an isolated action. Rather, it is a simultaneous suppression of the persistent inflammatory response that drives remodeling. This synergistic action signifies that SOP concurrently addresses both the “causes” (oxidative/immune dysregulation) and “consequences” (remodeling, RV dysfunction) of PH in a synergistic manner.

## 6. Limitations

Although this study confirms the protective effect of SOP against MCT‐induced PH—mitigating inflammation, apoptosis, and vascular remodeling—several limitations remain. First, the MCT model reflects toxin‐induced pathophysiology and fails to encompass the heterogeneity of human PH subtypes, potentially limiting its applicability to complementary models (Sugen/hypoxia models). Second, mechanistic conclusions rely solely on the pharmacological NRF2/HO‐1 inhibitor (ML385), lacking genetic validation (knockdown/rescue experiments, knockout models), NRF2 nuclear translocation data, and downstream antioxidant gene expression profiling (NQO1 and GCLC). Third, translational progress requires pharmacokinetic/pharmacodynamic studies, toxicity assessments, and formulation optimization, followed by clinical trials to validate efficacy and safety in human PH patients. We will address these gaps through targeted efforts to enhance research rigor and translational feasibility.

## 7. Conclusion

In summary, our research indicates that SOP is a promising multi‐target therapeutic agent for PH, potentially offering an integrated approach to PH treatment that combines vascular, immune, and cardiac protection.

## Author Contributions

Feng Xie and Jie Feng performed the experiments. Kai Li is in charge of picture processing. Yi Chen and Leilei Han interpreted the results. Yanqing Wu was responsible for the experimental conception and design.

## Funding

This work was supported by the Jiangxi Provincial Health Commission Science and Technology Innovation Key Project (Grant 2024ZD007), Jiangxi Province Key Laboratory of Molecular Medicine (Grant 2024SSY06231), and Jiangxi Provincial Traditional Chinese Medicine Science and Technology Program Project (Grant 2025020920).

## Disclosure

All authors reviewed or edited and approved the manuscript.

## Ethics Statement

Animal experiments are authorized by the Animal Care and Utilization Committee of Nanchang University (NCULAE‐20221031163).

## Consent

The authors have nothing to report.

## Conflicts of Interest

The authors declare no conflicts of interest.

## Supporting Information

Additional supporting information can be found online in the Supporting Information section.

## Supporting information


**Supporting Information** Raw images of representative Western blots – Representative catheter pressure traces – Full flow cytometry dot plots with gates – Example HE/Masson images with magnification and regions analyzed – Weekly weight monitoring of SD rats.

## Data Availability

Data will be made available upon request.
