# Supplementary material for: Targeting Pulmonary Hypertension: Elucidating Sophocarpine’s Protective Role via Preclinical Models
Source: Mediators Inflamm. 2026 Feb 23;2026:5524066. doi: 10.1155/mi/5524066 (PMC12927990; doi:10.1155/mi/5524066)
Supplement: Supplementary file 1 — Supporting Information Raw images of representative Western blots – Representative catheter pressure traces – Full flow cytometry dot plots with gates – Example HE/Masson images with magnification and regions analyzed – Weekly weight monitoring of SD rats. [file MI-2026-5524066-s001.docx]

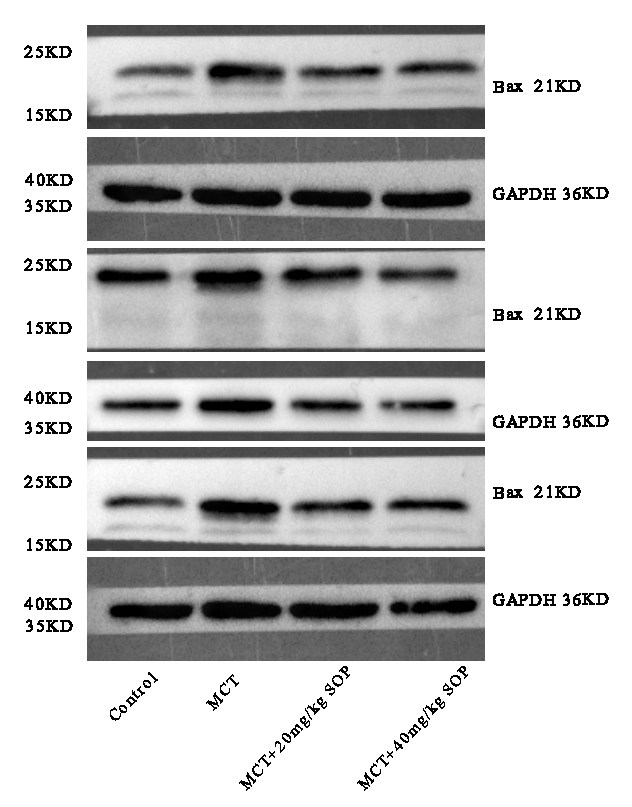
**Bax western blotting bands**

**BCL-2 western blotting bands**


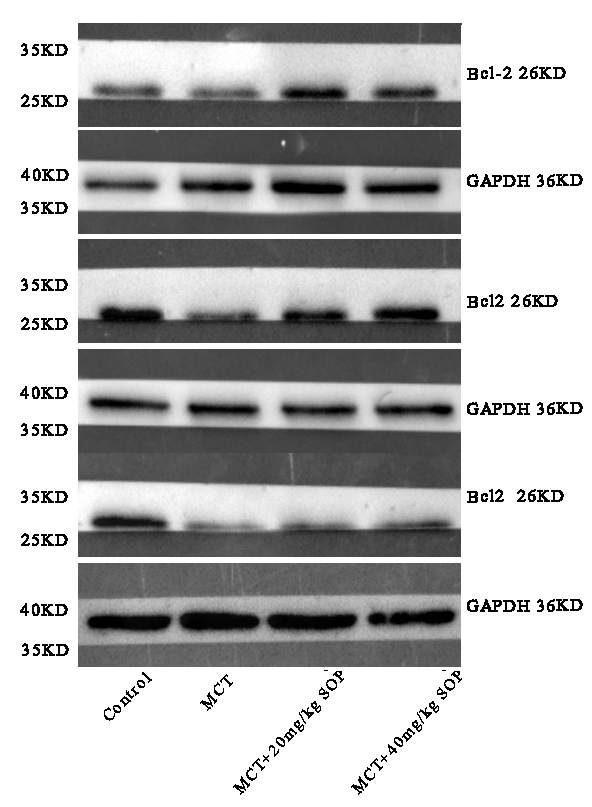


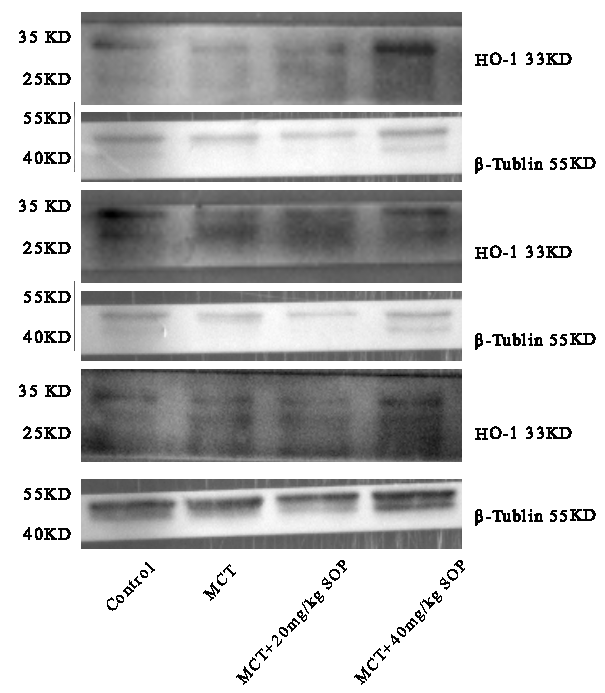
**HO-1 western blotting bands**

**iNOS western blotting bands**


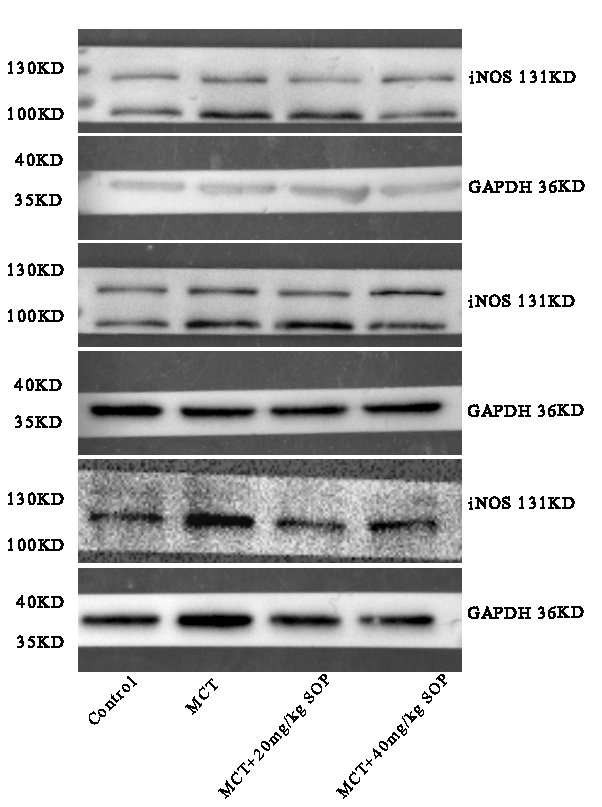


**IL-1β western blotting bands**


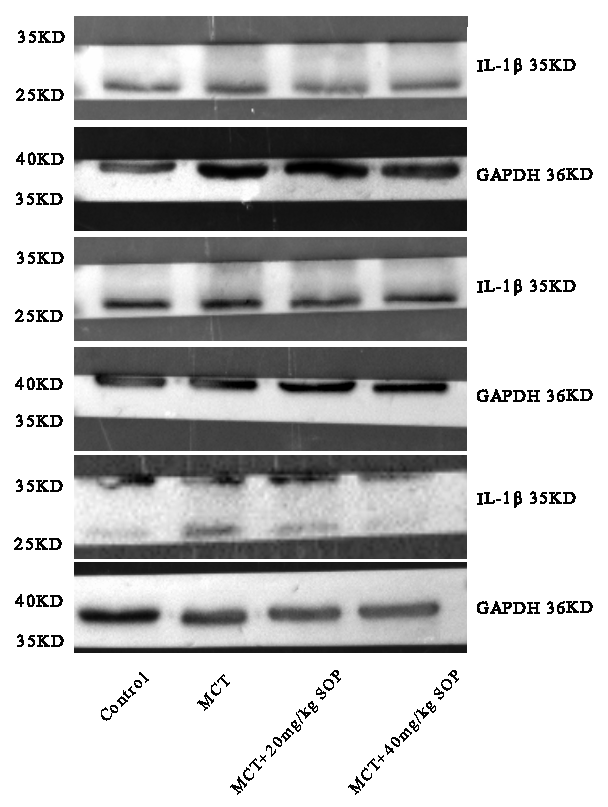


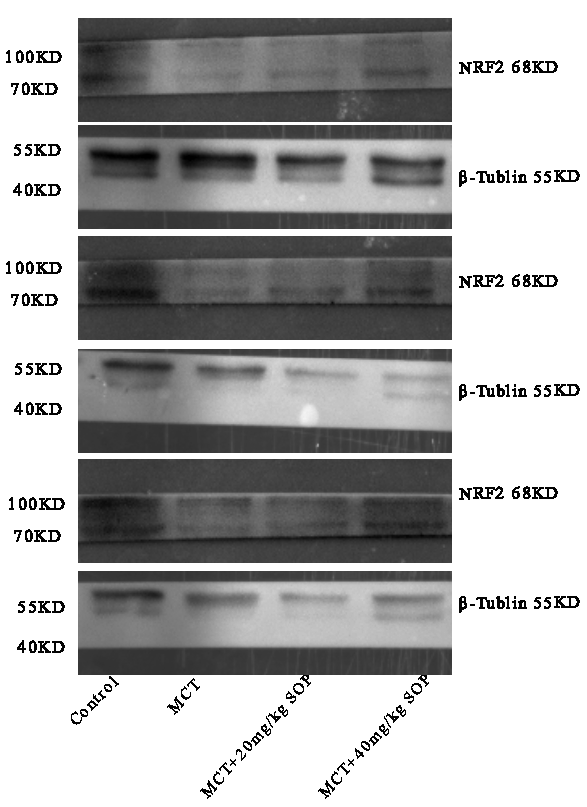
 **NRF2 western blotting bands**

**SOD-1 western blotting bands**


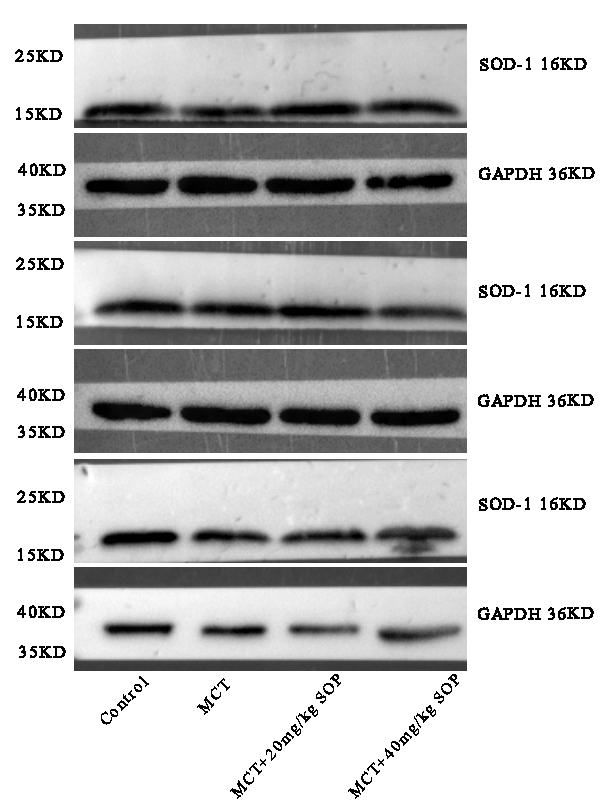


**TLR4 western blotting bands**


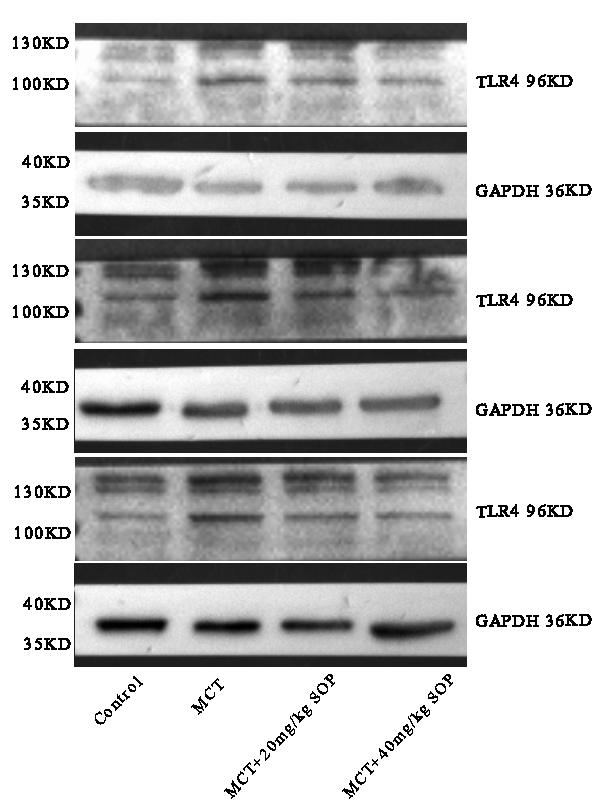


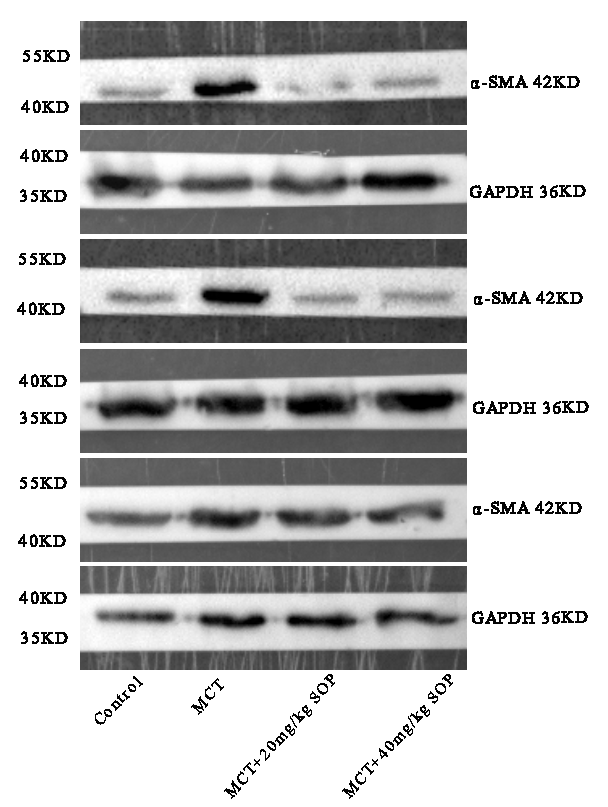
**α-SMA western blotting bands**


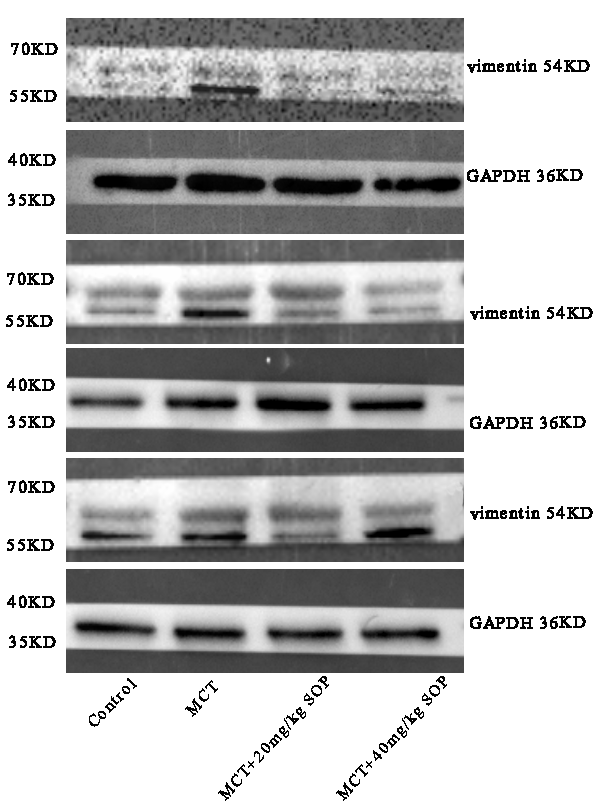
**Vimentin western blotting bands**

**representative catheter pressure traces**


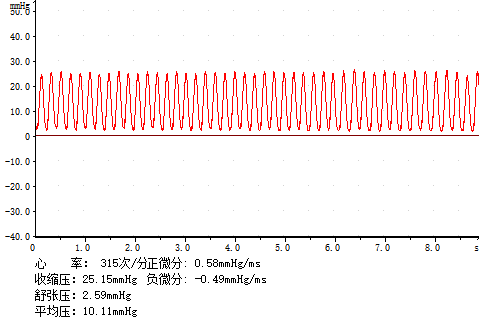


**
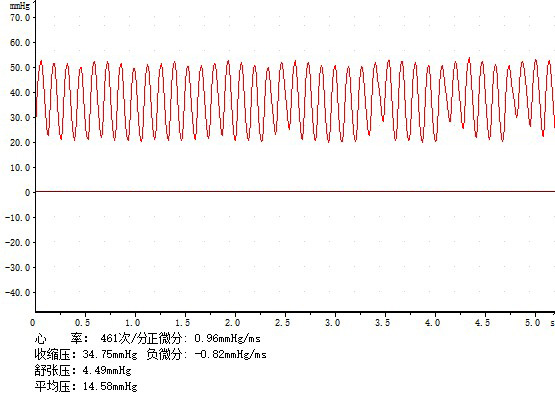
**


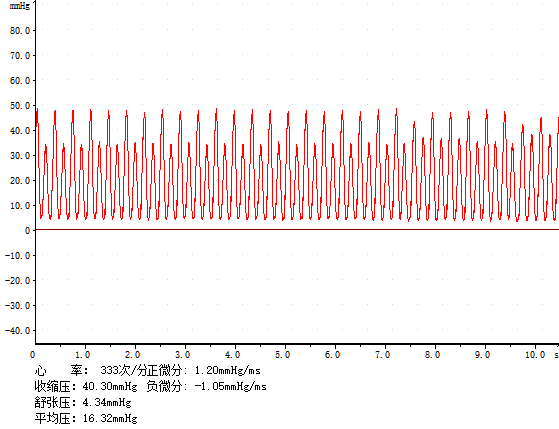


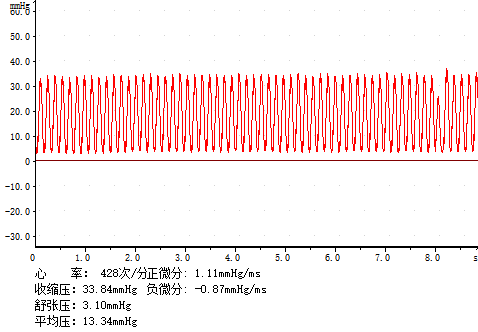


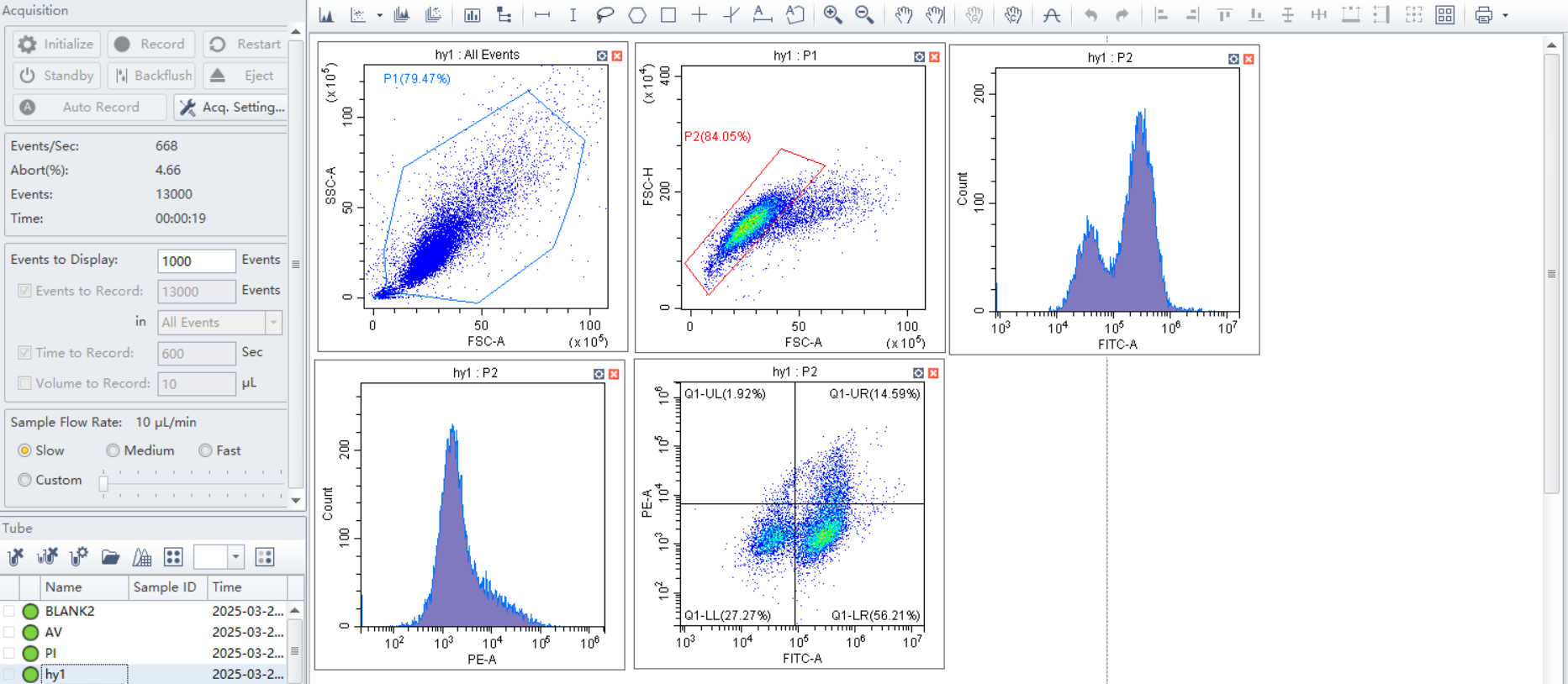
**full flow-cytometry dot plots with gates**

**example HE/Masson images with the magnification and regions analyzed.**

**HE heart Control group (Scale bar: 50μm, Magnification: 200X )**

**
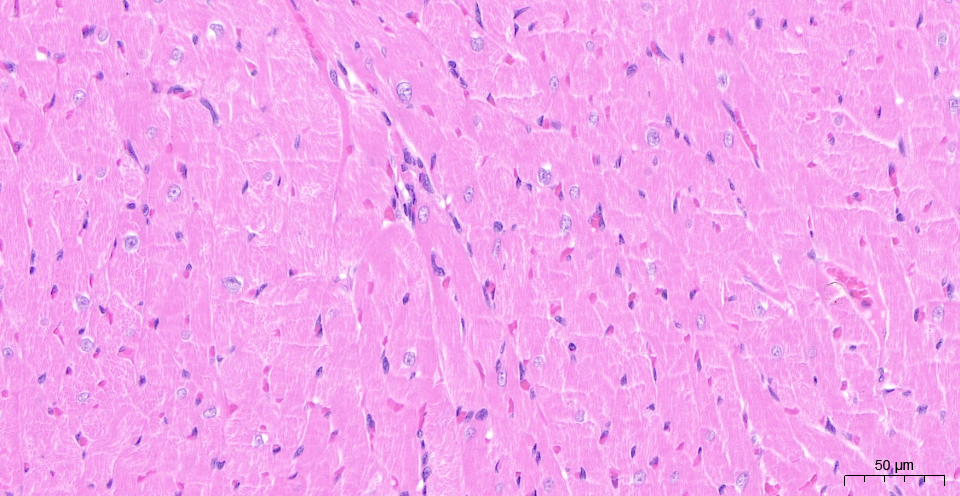
**

**MCT group**

**
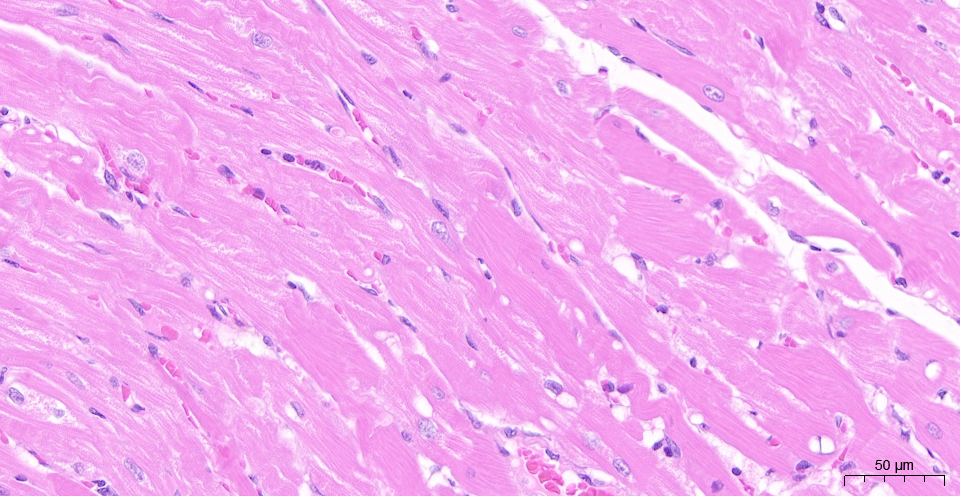
**

**MCT +20mg/kg SOP group**

**
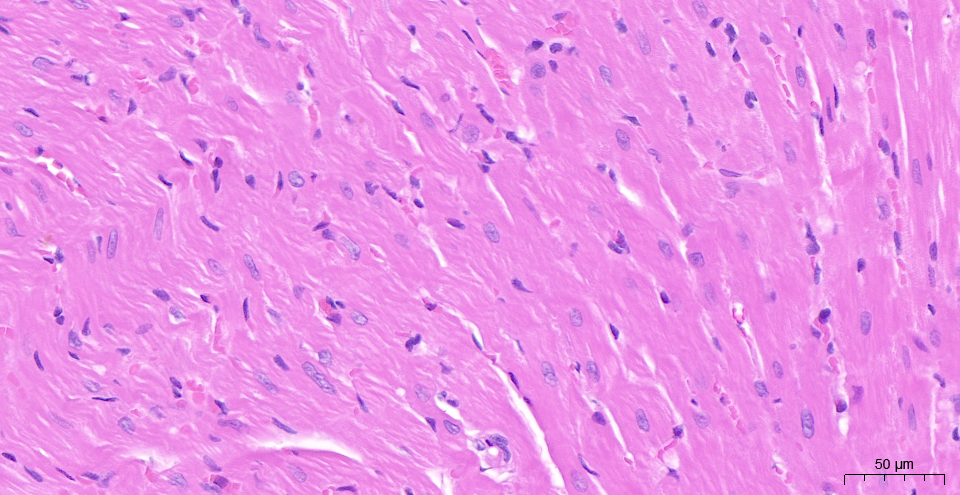
**

**MCT +40 mg/kg SOP group**

**
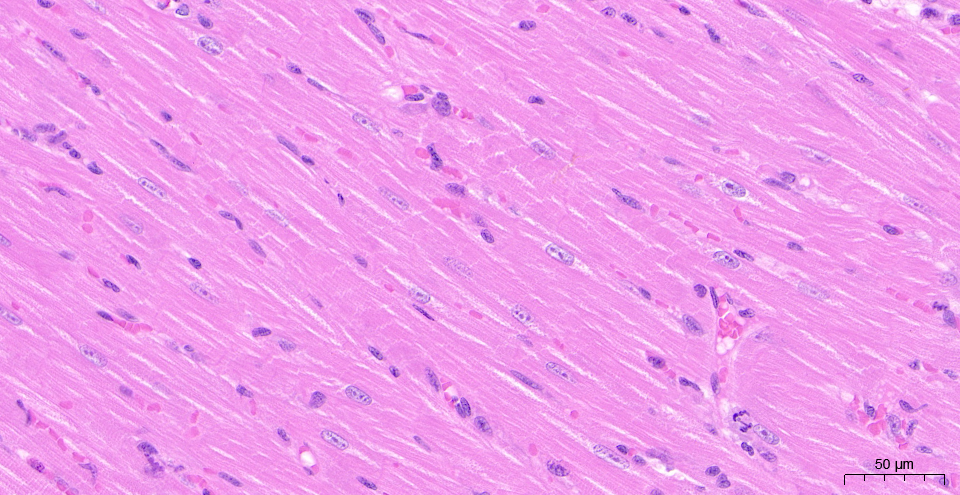
**

**Weekly weight monitoring of SD rats**

**Experiment Name:** Targeting pulmonary hypertension: Elucidating Sophocarpine’s Protective Role via Preclinical Models

**Rat Strain:** SD Rat

Monitoring Frequency: Once a week, at a fixed time (9:00-11:00 AM every Monday)

| Group | Rat ID | Week 1 (Acclimation) | Week 2 | Week 3 | Week 4 | Week 5 | Week 6 |
| --- | --- | --- | --- | --- | --- | --- | --- |
| Control Group | 1 | 198.5 | 221.5 | 245.3 | 268.7 | 292.4 | 315.8 |
|  | 2 | 205.3 | 227.8 | 251.6 | 275.2 | 298.9 | 322.5 |
|  | 3 | 189.2 | 211.4 | 233.8 | 256.1 | 278.5 | 301.2 |
|  | 4 | 212.6 | 235.1 | 258.7 | 282.3 | 305.9 | 329.6 |
|  | 5 | 201.8 | 224.2 | 246.9 | 269.5 | 293.1 | 316.7 |
|  | 6 | 195.7 | 218.3 | 240.9 | 263.5 | 286.1 | 308.7 |
|  | 7 | 208.4 | 231.0 | 253.6 | 276.2 | 298.8 | 321.4 |
|  | 8 | 187.9 | 210.5 | 233.1 | 255.7 | 278.3 | 300.9 |
|  | 9 | 210.5 | 233.1 | 255.7 | 278.3 | 300.9 | 323.5 |
|  | 10 | 197.3 | 219.9 | 242.5 | 265.1 | 287.7 | 310.3 |
| MCT  (60mg/kg) | 11 | 192.4 | 205.4 | 211.4 | 209.4 | 203.4 | 200.4 |
|  | 12 | 203.1 | 215.6 | 220.6 | 223.6 | 218.6 | 214.6 |
|  | 13 | 188.7 | 200.2 | 204.7 | 202.2 | 196.8 | 193.5 |
|  | 14 | 210.2 | 222.7 | 227.2 | 224.7 | 219.3 | 215.1 |
|  | 15 | 196.5 | 209.0 | 213.5 | 211.0 | 205.6 | 202.3 |
|  | 16 | 207.8 | 220.3 | 224.8 | 222.3 | 216.9 | 212.7 |
|  | 17 | 185.3 | 197.8 | 202.3 | 199.8 | 194.4 | 191.1 |
|  | 18 | 199.6 | 212.1 | 216.6 | 214.1 | 208.7 | 205.4 |
|  | 19 | 204.9 | 217.4 | 221.9 | 219.4 | 214.0 | 209.8 |
|  | 20 | 191.8 | 204.3 | 208.8 | 206.3 | 200.9 | 197.6 |
| MCT+SOP  20mg/kg | 21 | 195.6 | 213.6 | 226.6 | 235.6 | 239.6 | 241.6 |
|  | 22 | 208.2 | 225.7 | 237.7 | 245.7 | 248.7 | 249.7 |
|  | 23 | 187.5 | 204.5 | 216.5 | 224.5 | 227.5 | 228.5 |
|  | 24 | 211.3 | 228.8 | 240.8 | 248.8 | 251.8 | 252.8 |
|  | 25 | 198.7 | 215.7 | 227.7 | 235.7 | 238.7 | 239.7 |
|  | 26 | 204.5 | 221.5 | 233.5 | 241.5 | 244.5 | 245.5 |
|  | 27 | 189.8 | 206.8 | 218.8 | 226.8 | 229.8 | 230.8 |
|  | 28 | 201.2 | 218.2 | 230.2 | 238.2 | 241.2 | 242.2 |
|  | 29 | 207.6 | 224.6 | 236.6 | 244.6 | 247.6 | 248.6 |
|  | 30 | 193.4 | 210.4 | 222.4 | 230.4 | 233.4 | 234.4 |
| MCT+SOP  40mg/kg | 31 | 197.8 | 218.8 | 235.8 | 248.8 | 257.8 | 263.8 |
|  | 32 | 205.6 | 226.1 | 242.1 | 254.1 | 262.1 | 267.1 |
|  | 33 | 191.4 | 211.4 | 227.4 | 239.4 | 247.4 | 253.4 |
|  | 34 | 209.7 | 230.2 | 246.2 | 258.2 | 266.2 | 272.2 |
|  | 35 | 199.2 | 219.7 | 235.7 | 247.7 | 255.7 | 261.7 |
|  | 36 | 206.8 | 227.3 | 243.3 | 255.3 | 263.3 | 269.3 |
|  | 37 | 188.6 | 208.6 | 224.6 | 236.6 | 244.6 | 250.6 |
|  | 38 | 203.5 | 223.5 | 239.5 | 251.5 | 259.5 | 265.5 |
|  | 39 | 210.3 | 230.8 | 246.8 | 258.8 | 266.8 | 272.8 |
|  | 40 | 195.9 | 215.9 | 231.9 | 243.9 | 251.9 | 257.9 |
